# Long COVID clinical evaluation, research and impact on society: a global expert consensus

**DOI:** 10.1186/s12941-025-00793-9

**Published:** 2025-04-20

**Authors:** Andrew G. Ewing, David Joffe, Svetlana Blitshteyn, Anna E. S. Brooks, Julien Wist, Yaneer Bar-Yam, Stephane Bilodeau, Jennifer Curtin, Rae Duncan, Mark Faghy, Leo Galland, Etheresia Pretorius, Spela Salamon, Danilo Buonsenso, Claire Hastie, Binita Kane, M. Asad Khan, Amos Lal, Dennis Lau, Raina MacIntyre, Sammie McFarland, Daniel Munblit, Jeremy Nicholson, Hanna M. Ollila, David Putrino, Alberto Rosario, Timothy Tan, The Long COVID consensus expert panel (Table S1), David Joffe, Julien Wist, Yaneer Bar-Yam, Stephane Bilodeau, Jennifer Curtin, Rae Duncan, Mark Faghy, Leo Galland, Etheresia Pretorius, Danilo Buonsenso, Claire Hastie, Binita Kane, M. Asad Khan, Amos Lal, Dennis Lau, Sammie McFarland, Daniel Munblit, Jeremy Nicholson, David Putrino, Alberto Rosario, Lawrence B Afrin, Carlos Arturo, Nisreen Alwan, Sampath Kumar, Harashen Ashraf, Veronica Athie Morales, Philip Atkinson, Robert Axtell, Cipatli Ayuzo, Alba Azola, James Bagley, Sergio Bagnato, Amitava Banerjee, John Barry, Daniela Bassi-Dibai, Anne Bheréur, Gregory Bix, Jose-Ramon Blanco, Svetlana Blitshteyn, Hector Bonilla, Angela Bowers, Anna E.S. Brooks, Marie Bruyneel, Alexandra Brugler Yonts, Gunnar Bücker, Kathleen Capaccione, Harriet Carroll, Pascal Cathebras, Ashish Chaudhry, Bela Cheda, Karin Chia, Pratima Chowdary, Tae Hwan Chung, Leslie Conner, Caterina Conte, Caroline Dalton, Kara Darling, Helen Davies, Kate Davies, David Davies-Payne, Kevin Deans, Luis Orantes Del Carpio, Anne Doherty, Theresa Dowell, Raechel A Evans, Andrew Ewing, Emilia Liana Falcone, Bingwen Eugene Fan, Emily Fatakhov, Kelly Fearnley, Artur Fedorowski, Carlo Ferrarese, Francesco Ferraro, Gemma Figtree, Laurie Findlay, Alberto Fortini, Matteo Foschi, Francois Gagnon, Nick Gall, Natasha Gerbis, Gholamrezanezhad Ali, Amine Ghram, Andréa Lúcia da Silva Goncalves, Helen Goss, Stephanie Grach, Caroline Gregoire, Gary Groot, Riccardo Guanà, Eric Guedj, Merel Hellemons, Melanie Hoppers, Mohammad-Salar Hosseini, Lee Ingle, Linn Jarte, Leslie Kasza, David Kaufman, Sundeep Kaul, Douglas Kell, Daphne Kemp, Robin Kerr, Abbas Khushnood, SangYun Kim, Cheryl Koh, Nik Kudiersky, Katharina Kurz, Gonzalo Labarca, Francisco Landi, Jaco Laubscher, Joshua Leisk, Jacob Leone, Graham Lloyd-Jones, C Raina MacIntyre, Kushal Madan, Stuart Malcolm, Harsha Master, Gez Medinger, Yolanda Meije, Alexios-Fotios Mentis, Francisco Mera-Cordero, David Miller, Bethan Myers, Hiten Naik, Javaid Nauman, Josef Niebauer, Estefania Nova-Lamperti, Maria Olivier, Otieno Martin Ong’wen, Rebecca Owen, Kelly O’Brien, Stefano Pallanti, Roger Paredes, Tina Peers, Alice Perlowski, Colin Pidgeon, Julianne Pitzele, Chris Ponting, Anna Porter, Amy Proal, Milo Puhan, Shaun Peter Qureshi, Sunil Raina, Nandini Raj, Ashwin Rajenesh, Clare Rayner, Alfonso J Rodriguez-Morales, Luca Roncati, Cilla Rosen, Ilene Ruhoy, Leslie Rydberg, Špela Šalamon, Carmen Scheibenbogen, Sarah Schenck, Katharine Seagly, Jaffer Shah, Ben Sinclair, Ioakim Spyridopoulos, Warren Tate, Claire Taylor, Catherine Tewolde, Alain Thierry, Callum Thomas, Tracey Thompson, Irene Tosetti, Eleonora Trecca, Jordan Vaughn, Chantelle Venter, Amber Vermeesch, Joe Vipond, Guiulia Vivaldi, Maxine Waters, William Weir, Emma Weisblatt, May Wong, Rob Wüst, Azfar Zaman, James Zhang

**Affiliations:** 1 Department of Chemistry and Molecular Biology, University of Gothenburg, Gothenburg, Sweden; 2 World Health Network Long Covid Expert Advisory Group, Cambridge, USA MA; 3 Respiratory and Sleep Medicine, Royal North Shore Hospital, St Leonards, Australia; 4 Woolcock Institute of Medical Research (Sleep Group), Sydney, Australia; 5 Department of Neurology, University at Buffalo Jacobs School of Medicine, Buffalo, NY USA; 6 Dysautonomia Clinic, Williamsville, NY USA; 7 Liggins Institute, The University of Auckland, Auckland, New Zealand; 8 School of Biological Sciences, Faculty of Science, The University of Auckland, Auckland, New Zealand; 9 The Maurice Wilkins Centre, Auckland, New Zealand; 10 Australian National Phenome Centre, Murdoch University, Murdoch, Australia; 11 Imperial College London, London, UK; 12 Chemistry Department, Universidad del Valle, Cali, Colombia; 13 New England Complex Systems Institute, Cambridge, MA USA; 14 Department of Bioengineering, McGill University, Montreal, Canada; 15 Real Time Health Monitoring, San Francisco, CA USA; 16 The Newcastle Hospitals NHS Foundation Trust, Newcastle Upon Tyne, UK; 17 Biomedical and Clinical Exercise Science Research Theme, University of Derby, Derby, UK; 18 Foundation for Integrated Medicine, New York, NY USA; 19 Department of Physiological Sciences, Faculty of Science, Stellenbosch University, Stellenbosch, Western Cape South Africa; 20 Department of Biochemistry, Cell and Systems Biology, Institute of Systems, Molecular and Integrative Biology, Faculty of Health and Life Sciences, University of Liverpool, Liverpool, UK; 21 Department of Woman and Child Health and Public Health, Fondazione Policlinico Universitario A. Gemelli IRCCS, Rome, Italy; 22 Founding Member, Long Covid Support, London, UK; 23 Manchester University Foundation Trust, School for Biological Sciences, University of Manchester, Manchester, UK; 24 Directorate of Respiratory Medicine, Manchester University Hospitals, North West Lung Centre, Manchester, M23 9LT UK; 25 Division of Pulmonary, Critical Care and Sleep Medicine, Mayo Clinic, Rochester, MN USA; 26 The University of Adelaide and Royal Adelaide Hospital, Adelaide, SA Australia; 27 Biosecurity Program, Kirby Institute, University of New South Wales, Sydney, Australia; 28 CEO & Founder Long Covid Kids, London, UK; 29 Imperial College and King’s College, London, UK; 30 Australian National Phenome Centre, Murdoch University, Perth, WA Australia; 31 Faculty of Health and Medical Sciences, University of Western Australia, Crawley, Australia; 32 Imperial College London, London, UK; 33 Nanyang Technological University, Singapore, Singapore; 34 Regional Adjunct Professor, Khon Kaen University, Khon Kaen, Thailand; 35 Institute for Molecular Medicine Finland, FIMM, HiLIFE, University of Helsinki, Helsinki, Finland; 36 Broad Institute of Harvard and MIT and Center of Genomic Medicine, Massachusetts General Hospital, Boston, MA USA; 37 Department of Anesthesia, Critical Care and Pain Medicine, Massachusetts General Hospital, Boston, MA USA; 38 Centre for Genomic Medicine, Massachusetts General Hospital, Harvard Medical School, Boston, MA USA; 39 Cohen Center for Recovery From Complex Chronic Illness, Department of Rehabilitation and Human Performance, Icahn School of Medicine at Mount Sinai, New York, NY USA; 40 Infection Prevention Team, World Health Network, Cambridge, MA USA; 41 Consultant Cardiologist, Westmead and Blacktown Hospitals, Sydney, Australia; 42 Conjoint Professor, School of Medicine, Western Sydney University, Sydney, Australia; 43 Conjoint Clinical Associate Professor Sydney Medical School, Sydney University, Sydney, Australia; 44 Adjunct Associate Professor, School of Medical Sciences, Faculty of Medicine, University of New South Wales, Sydney, Australia; 45 Area Pediatrica, Dipartimento di Scienza Della Vita e Sanità Pubblica, Università Cattolica del Sacro Cuore, Rome, Italy; 46 Department of Paediatrics and Paediatric Infectious Diseases, Institute of Child’s Health, Sechenov First Moscow State Medical University (Sechenov University), Moscow, Russia

**Keywords:** Long COVID, Definition, Diagnosis, Treatment, Research, Societies

## Abstract

**Background:**

Long COVID is a complex, heterogeneous syndrome affecting over four hundred million people globally. There are few recommendations, and no formal training exists for medical professionals to assist with clinical evaluation and management of patients with Long COVID. More research into the pathology, cellular, and molecular mechanisms of Long COVID, and treatments is needed. The goal of this work is to disseminate essential information about Long COVID and recommendations about definition, diagnosis, treatment, research and social issues to physicians, researchers, and policy makers to address this escalating global health crisis.

**Methods:**

A 3-round modified Delphi consensus methodology was distributed internationally to 179 healthcare professionals, researchers, and persons with lived experience of Long COVID in 28 countries. Statements were combined into specific areas: definition, diagnosis, treatment, research, and society.

**Results:**

The survey resulted in 187 comprehensive statements reaching consensus with the strongest areas being diagnosis and clinical assessment, and general research. We establish conditions for diagnosis of different subgroups within the Long COVID umbrella. Clear consensus was reached that the impacts of COVID-19 infection on children should be a research priority, and additionally on the need to determine the effects of Long COVID on societies and economies. The consensus on COVID and Long COVID is that it affects the nervous system and other organs and is not likely to be observed with initial symptoms. We note, biomarkers are critically needed to address these issues.

**Conclusions:**

This work forms initial guidance to address the spectrum of Long COVID as a disease and reinforces the need for translational research and large-scale treatment trials for treatment protocols.

**Supplementary Information:**

The online version contains supplementary material available at 10.1186/s12941-025-00793-9.

## Introduction

The World Health Organisation (WHO) lists ‘confirmed’ cases of COVID-19 at 775 million [1]. This number is likely much greater due to the limitations of testing and very limited surveillance [2]. Following acute COVID-19, the risk of developing symptoms that last beyond the initial illness, is estimated to be 15% per individual per infection [2]. This ailment is often termed Long COVID, but has several names including post-COVID conditions (PCC), post-acute COVID-19 or post-acute sequelae of SARS-CoV-2 infection (PASC).

Defining, diagnosing, treating, and understanding Long COVID and its impact on society pose some of the most significant scientific and medical questions of our time. The true global prevalence of Long COVID is likely much higher than 100 million as risk is amplified by reinfection [3] similarly affecting all ethnicities, with most cases in 18- to 64-year-olds. Women are affected approximately twice as often as men [4]. SARS-CoV-2 can also cause organ damage in individuals both with and without symptoms [5, 6].

From July 2023 to February 2024, a global panel of experts of more than half physicians diagnosing and treating Long COVID, as well as researchers and those with lived experience, engaged in a modified Delphi consensus process [7, 8]. Topics included definition, diagnosis and clinical assessment, treatment, research, and socioeconomic factors related to Long COVID. The overarching goal was to provide recommendations to physicians, researchers and policy makers.

## Methods

### Delphi expert panel member selection

This modified Delphi electronic survey (using the Survey Monkey platform) on Long COVID was conducted under the governance and oversight of the World Health Network (WHN). We used an open sampling approach to generate the panel for this Delphi study. The WHN Long COVID working group, with a membership of 12 members, initiated the study. During the progression of the study the group enlarged to 20 members and an additional 14 members were recruited to form an extended evaluation committee. The working group met bi-weekly and the extended committee on invitation. The target panel for the survey was chosen to include clinicians (e.g., general practitioners, pulmonologists, cardiologists, neurologists) and researchers with expertise in Long COVID. A comprehensive literature search was carried out to identify physicians and researchers working and publishing on Long COVID and an email inquiry was sent to them (n = 1574) to ascertain their willingness to participate. As new literature was published, new members were invited at each new round in the survey.

**Fig. 1:**
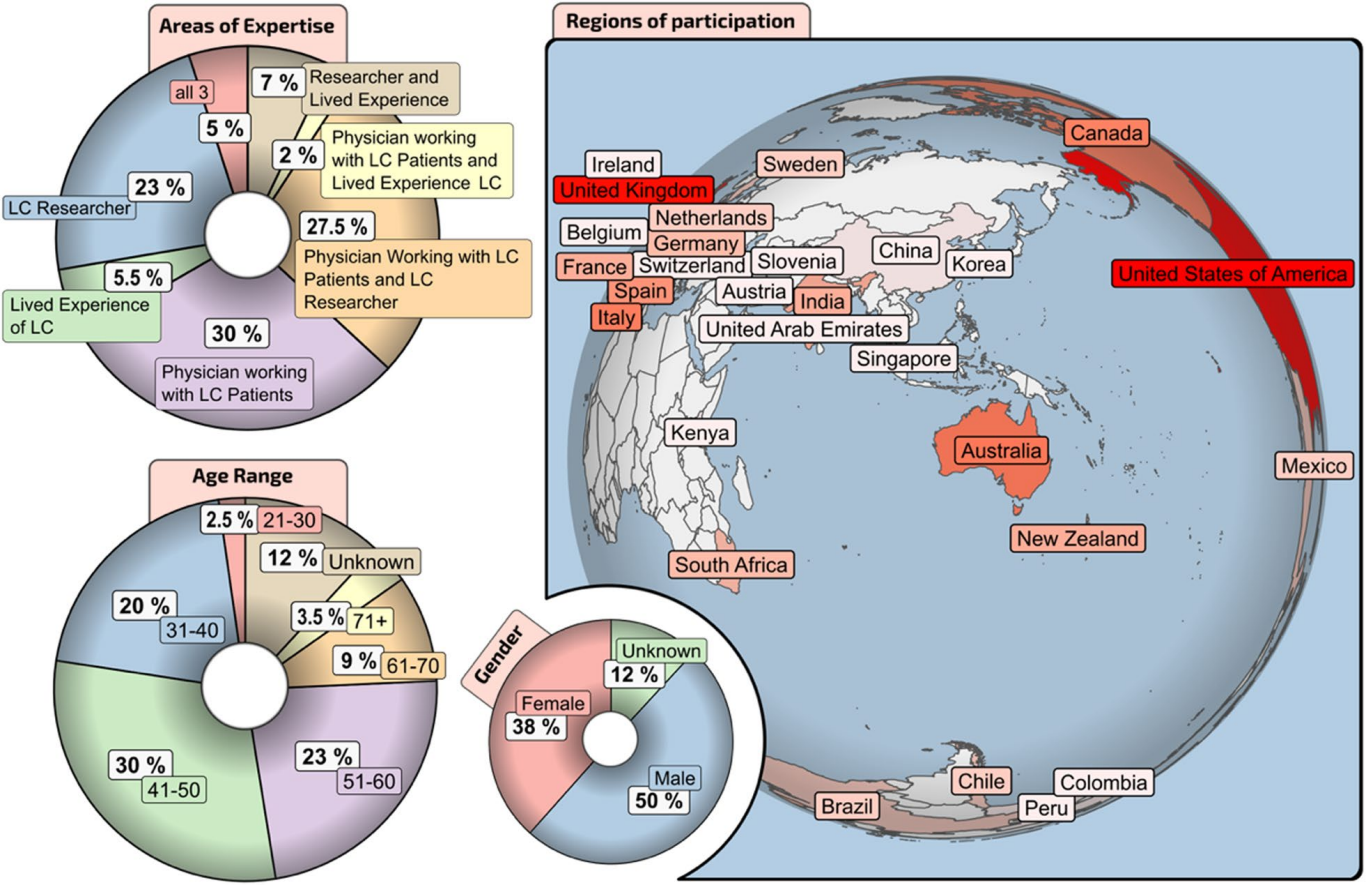
Breakdown of Long COVID consensus expert panel giving areas of expertise, age range, gender pattern, and countries

Areas of expertise, 28 countries of participation, and panel genders and age groups are shown in Fig. 1. Selection of the expert panel involved semi-purposeful sampling [9], which had the criterion that panellists had expertise in COVID-19/Long COVID and/or rehabilitation.

### Delphi method data collection

The Delphi method was modified to ensure the methodology was suitable for the study aims instead of configuring the study aims to fit the methodology [10, 11]. The first round of a traditional Delphi typically uses open questioning to identify the focus. However, in the present study, this was modified to include 34 health care professionals as well as patients and public involvement and engagement (PPIE), reviewing the existing literature until July 2023, and generating structured questions using a roundtable approach.

**Fig. 2:**
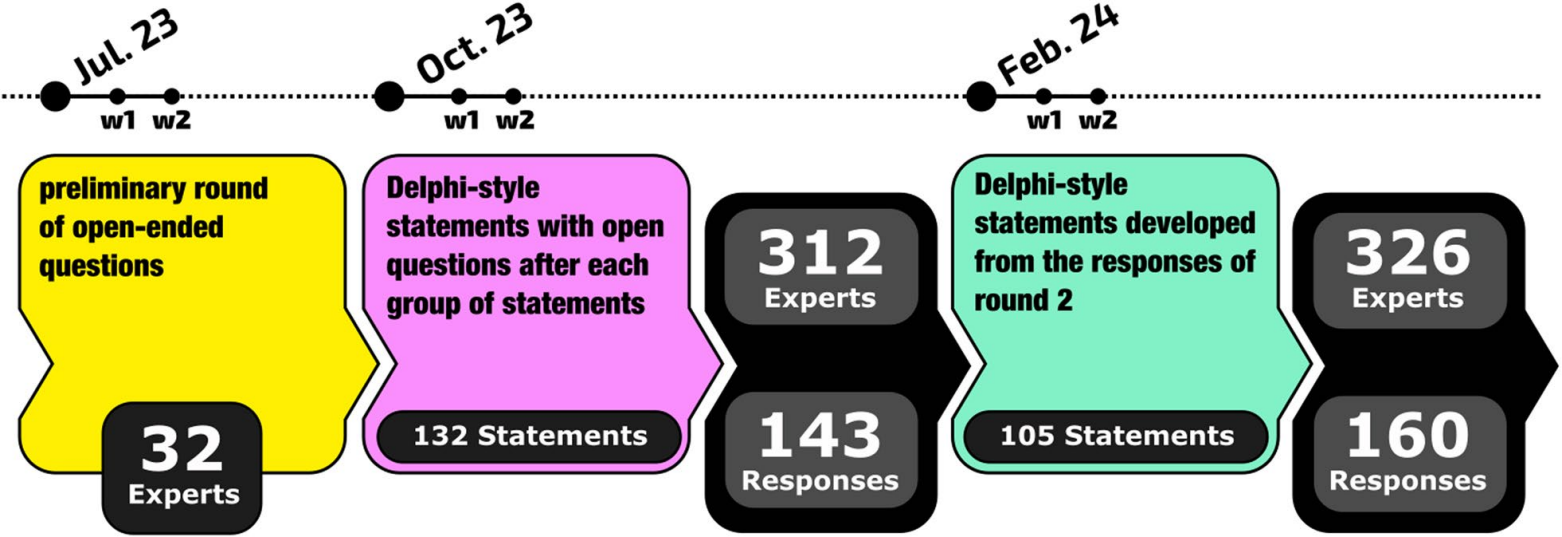
Design of the 3-stage modified Delphi survey. A total of 179 experts participated over the three stages. A total of 1574 experts from searching the literature were initially contacted and 32 gave responses to an open-ended set of questions (see Table S4). In the Delphi rounds, the response rate to the first round of 132 statements was 46% and the response rate to the second round of 105 statements was 49%

Figure 2 shows a breakdown of the survey process and numbers of responses. The international surveys included a preliminary round of open-ended questions (SI Table S4) to generate a broad range of opinions and perspectives. This provided 32 responses that were then used by the working group to generate Delphi-style statements in the areas of Long COVID definition, diagnosis, treatment, evaluation of treatment, research, and social issues (SI Table S5), with open questions after each group of statements for round 2 of the survey. Open-text responses were not analysed using a formal process but were considered by the trial steering group using a roundtable approach with individual members adding potential new statements for discussion and refinement by the group. The third round of the survey (SI Table S6) was developed from the responses from round 2. These statements were developed by the extended committee to A) modify statements not receiving consensus in the earlier round and B) to address the open-ended comments. Respondents were given ∼3 weeks to complete the surveys.

### Delphi data analysis

A 5-point Likert Scale was used (Tables S5 and S6) and for some statements ‘do not have the expertise to answer’ was considered necessary based on the diversity of the expert panel. Anonymized results were summarised into excel tables and a sum for agree and strongly agree was determined for consensus. The proportion who chose ‘I do not have the expertise to answer’ was removed from the denominator to calculate levels of agreement/disagreement. A supermajority (that is, ≥ 67% combined agreement – strongly agree and agree) was used as a minimum cut-off for consensus. This more demanding cut-off (versus a simple majority of greater than 50%) was decided to ensure clear consensus.

## Results

**Fig. 3:**
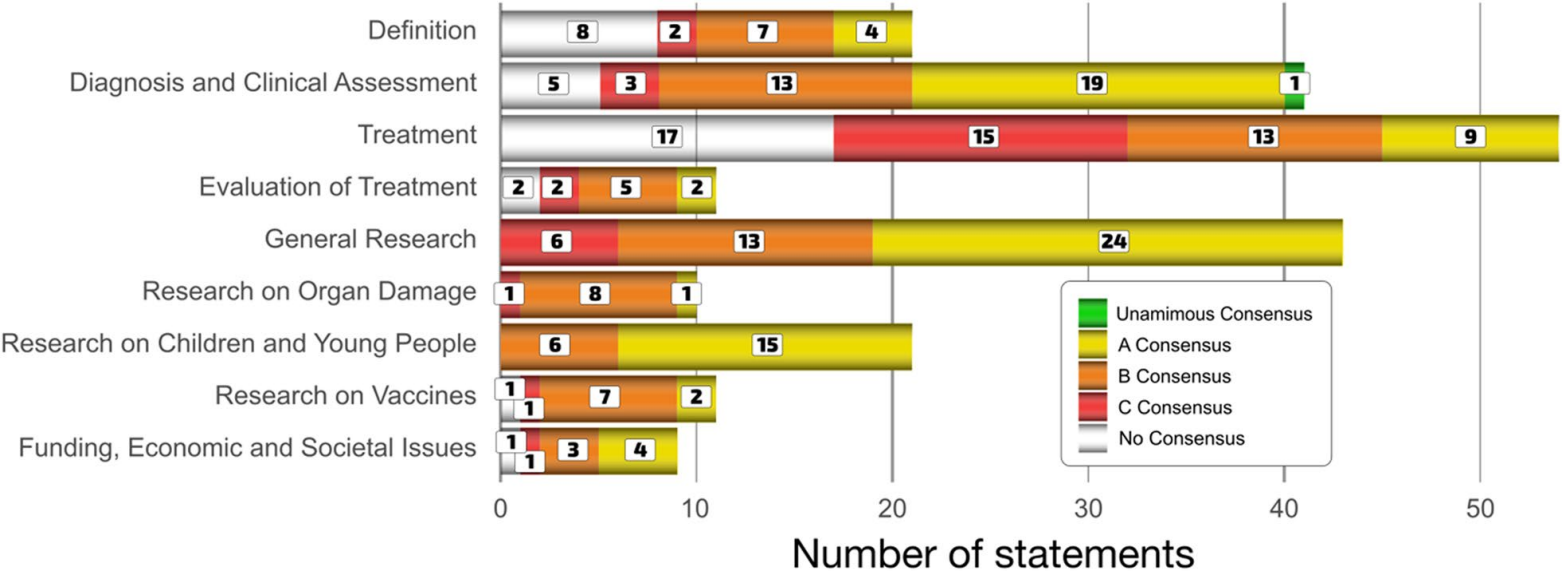
Summary of consensus statements by topic and consensus level (listed in Table 1)

A list of 187 statements reaching consensus by topic across survey rounds two and three is given in Table 1. A pictorial summary of consensus statement numbers by topic is given in Fig. 3. Statements are arranged with the following levels of agreement: ‘U’ denotes unanimous (100%) agreement; ‘A’ denotes 90–99% agreement; ‘B’ denotes 78–89% agreement; ‘C’ denotes 67–77% agreement.

**Table 1:** Statements reaching consensus by topic and level of agreement

	Cat.: Definitions
Consensus level A statements	The Long COVID definition should also include a criterion for significant functional impairment from baseline (including reduction in effort tolerance, even without additional new symptoms)
	Acknowledging that many clinical phenotypes of Long COVID align with established syndromes such as post-intensive care syndrome, ME/CFS, and POTS, it is important to recognize that these conditions may have distinct pathophysiologic mechanisms
	Long COVID is evident in young individuals with a documented history of SARS-CoV-2 infection, displaying at least one enduring physical, cognitive, or neuro-psychiatric symptom that persists for a minimum of 8–12 weeks after the initial infection, where other causes have been excluded
	It is important to recognize that Long COVID is an umbrella term encompassing several different disorders including e.g. dysautonomia, neuroinflammation, endothelial dysfunction, hypercoagulation, impaired fibrinolysis, mast cell disorders, and mitochondrial dysfunction. It is therefore important to establish a minimum Long COVID diagnostic workup for these conditions
Consensus level B statements	As Long COVID is a broad and inclusive term, it is valuable to create subcategories based on phenotype/endotype. It should be understood that many long-term pathophysiological outcomes of SARS CoV-2 infections may not directly result in specific symptoms, but may still have long-term consequences (e.g. cardiovascular risks) that require biochemical, immunological or metabolic
	assessment. This is an important limitation of questionnaire-based studies in relation to clinically actionable Long COVID definitions. Nonetheless, any physiological metrics are still real components of the phenotype or endotype even if they are not measured directly
	The term Long COVID should be used for those that have persistent or relapsing and remitting functional impairment. Functional impairment could also include asymptomatic components
	Long COVID is triggered by SARS-CoV2 infection; the resulting pathophysiology may include immune-mediated multi-organ inflammatory changes, endothelial dysfunction, hypercoagulability, micro-clotting, and downstream effects on mitochondrial dysfunction
	Long COVID includes any new symptoms with onset after acute COVID-19, whether those symptoms appear during the acute infection or have their onset within a period of 12 weeks after the acute infection and persist for at least 8 weeks
	Long COVID is characterized by the persistence of symptoms and signs for at least 3 months after the onset of initial symptoms or a recurrence within 2 months after the resolution of acute COVID-19. The evolving nature of symptoms during the course of the illness contributes to a reduction in functional capacity and overall health compared to the pre-infection state, potentially exacerbating pre-existing health conditions. Pre-existing conditions (especially those of an inflammatory nature) are likely to interact with the Long COVID phenotype, and the same would be expected for newly acquired conditions, including SARS CoV-2 reinfections or other viral diseases, this in turn will impact on the expressed Long COVID phenotype
	Long COVID includes impairment in any area of function e.g. physical or cognitive, that follows acute COVID-19 and persists for at least 8–12 weeks, independent of symptoms
	Long COVID is not a singular disease entity; it comprises distinct yet related virally triggered host response pathways. Proper diagnosis is crucial, considering conditions like virally induced POTS, MCAS, acquired vascular phenotype, post-COVID-19 neuroinflammation, and post-COVID-19 myocarditis. Recognizing each diagnosis separately is essential as treatments differ with each
Consensus level C statements	Long COVID best describes the persistence of symptoms that started with COVID-19 and have failed to resolve 8 weeks after the onset of the illness
	The WHO defines Long COVID as “the continuation or development of new symptoms 3 months after the initial SARS-CoV-2 infection, with these symptoms lasting for at least 2 months with no other explanation. "This is a valid definition of Long COVID
	Cat.: Diagnosis and clinical assessment
Consensus level U statements	There is a need to educate all health care workers about the possible complications following SARS-CoV-2 infection and to ensure that patients are listened to, appropriately investigated and supported
Consensus level A statements	A functional ability/capacity evaluation is important in the assessment of Long COVID
	It is important to take into account a person’s self-reported cognitive difficulties even if their performance on standard cognitive tests is within normal limits
	Clinical assessment of Long COVID should examine the change in a person’s functional abilities from their previous baseline, as well as the cost of maintaining functioning (e.g. more effort and/or time required, and physical or cognitive exhaustion following tasks)
	Screening for organ and circulatory dysfunction is important in the assessment and management of Long COVID
	In diagnosing Long COVID patients it is important to screen for general symptoms including fatigue, post-exertional malaise (PEM)/post exertional symptom exacerbation (PESE), and persistent fevers
	In diagnosing Long COVID patients it is important to assess neurological/brain symptoms including sleep disruption, headache, seizures, cognitive dysfunction, mood changes, sensitivity to stimuli of the senses, hearing, vertigo, loss of smell/taste, dry eyes or mouth, temperature dysregulation, paraesthesia, changes in sweating, syncope, tremor/internal vibrations, hallucinations, depression and anxiety
	In diagnosing Long COVID patients it is important to assess cardiorespiratory symptoms including dizziness on standing, palpitations, chest pain, SOB, cough, wheezing, and tachycardia with modest exertion
	In diagnosing Long COVID patients it is important to assess vascular symptoms including limb pain or heaviness, nail changes, Raynaud’s syndrome, hair loss, blotchy skin, vascular rash, and COVID-19 toes
	In diagnosing Long COVID patients it is important to assess gastrointestinal symptoms including changes in bowel habit, abdominal pain, nausea, bloating, food regurgitation or vomiting, new food intolerances, and changes in weight
	In diagnosing Long COVID patients it is important to screen for orthostatic intolerance and dysautonomia, including postural orthostatic tachycardia syndrome (POTS)
	Evaluation of all aspects of Long COVID should account for the fluctuating nature of many symptoms and recognise the need for repeated measurements to capture clinical findings (e.g. autonomic dysfunction, cognitive difficulties)
	In clinical assessment of Long COVID, it is important to evaluate for sleep disturbances
	It is important that clinicians realise that most standard screening tests will come back normal and specific tests are needed for diagnosis of various pathologies in Long COVID
	Diagnostic investigations should be tailored to the symptoms of each patient
	Clinically validated Long COVID-specific biomarkers, when available, will play a role in diagnosing Long COVID, despite its complexity
	It is important to ask Long COVID patients about changes in their ability to perform activities of daily living (ADLs) to understand disease severity
	It is essential to screen for appropriate categories of cardiovascular disease relevant to a patient’s clinical presentation in the workup of Long COVID
	Evaluation for neuropsychiatric manifestations, such as anxiety, depression, sleep disturbance, cognitive disturbance, and/or ADHD should be part of a comprehensive assessment of Long COVID patients
	Careful assessment for autoimmune, cardiorespiratory, endocrine, and other post-COVID-19 complications across body systems is an important part of the management of Long COVID
Consensus level B statements	Validated questionnaires are important in the assessment of Long COVID
	Neurocognitive testing is important for the assessment of Long COVID
	Bedside tests to diagnose orthostatic intolerance are important in the assessment of Long COVID
	Screening for immune dysfunction/dysregulation is important in the assessment and management of Long COVID
	In diagnosing Long COVID patients it is important to assess allergy symptoms including hives, anaphylaxis, new onset or worsening of existing allergies, dermatographism, nasal congestion or rhinorrhoea, atopy, rashes, diarrhoea, and joint pain
	In diagnosing Long COVID patients it is important to assess genitourinary symptoms including sexual dysfunction, menstrual changes, loss of libido, urinary frequency, dysuria, and problems with bladder emptying
	In diagnosing Long COVID patients it is important to assess endocrine symptoms, for example weight loss/gain, tremors, polyuria/polydipsia (diabetes), premature menopause
	In assessing Long COVID patients it is important to use imaging studies for persistent and unexplained symptoms
	Novel tests for micro-clot formation need to be further validated and integrated into clinical use as potential vascular biomarkers of Long COVID
	Assessment of endothelial function and platelet hyperactivation should be routinely carried out in Long COVID patients as part of the workup and ongoing management when these tests become available to clinicians in the future
	In the clinical and diagnostic assessment of Long COVID pathophysiology, cardiopulmonary exercise testing (CPET) can be used to evaluate cardiorespiratory function and functional capacity in patients for whom the testing is safe and appropriate
	It is important to evaluate for symptoms of perimenopause, premature onset menopause and worsening of existing menopause in females as part of Long COVID evaluation, as there is some data showing that COVID-19 can trigger hormonal changes
	Screening for iron deficiency can be useful as part of a comprehensive assessment in Long COVID
Consensus level C statements	The diagnosis of Long COVID does not require that the person have laboratory confirmation of COVID-19 infection during a period of 4–8 weeks prior to the onset of symptoms
	Emergent biomarkers of neuroaxonal or glial fibrillary damage, such as NfL and GFAP, can be important in diagnosing Long COVID. (48 said they did not have the expertise)
	In diagnosing Long COVID, testing for thrombophilias or indicators for increased clotting risk (i.e. Factor V Leiden, anti-phospholipid antibodies, homocysteine, prothrombin, hemochromatosis testing, etc.) can be helpful
	Cat.: Treatment
Consensus level A statements	Multidisciplinary teams (MDTs) are a useful model for care of Long COVID
	In treating Long COVID it is important to consider treatment of orthostatic intolerance and dysautonomia, including postural orthostatic tachycardia syndrome (POTS)
	In treating Long COVID is important to treat sleep disturbances
	In treating Long COVID it is important to consider treatment of newly identified diabetes and/or dyslipidemia or consider modifying the treatment of pre-existing diabetes and/or dyslipidemia
	In treating Long COVID it is important to consider treatment of newly identified pain or consider modifying treatment of pre-existing pain
	In treating Long COVID it is important to consider the treatment of newly identified blood pressure abnormalities or consider modifying treatment of pre-existing abnormalities
	Treatments should be tailored to the history and clinical examination
	Psychological therapies can be useful in supporting the mental health of those with Long COVID in conjunction with treatments that target the pathophysiology
	Cognitive screening tools such as the Montreal Cognitive Assessment (MOCA) or the Mini Mental State Exam (MMSE) may not be appropriate for testing of cognitive disturbance in patients with Long COVID. More comprehensive cognitive testing, sometimes performed on more than one occasion, may be required to detect & assess the severity of cognitive dysfunction in Long COVID patients
Consensus level B statements	In treating Long COVID it is important to consider treatment of abnormal clotting pathology
	In treating Long COVID it is important to consider treatment of mast cell activation syndrome
	In treating Long COVID it is important to consider treatment of resting tachycardia
	In treating Long COVID it is important to consider treatment of myocarditis/pericarditis
	In treating Long COVID it is important to consider treatment of gut dysbiosis
	Cardiac or respiratory pathology should be ruled out before prescribing graded exercise therapy
	Drugs that modulate the autonomic nervous system (e.g. ivabradine, beta blockers, midodrine) can be useful in treating some Long COVID patients
	Non-opioid pain medications can be useful for treatment of e.g. small fibre neuropathy pain and headache in Long COVID
	Antidepressants can be useful in supporting the mental health of those with Long COVID where appropriate, and in conjunction with other treatments that target the pathophysiology
	Therapies directed at endothelilitis or endothelial injury are useful in treating Long COVID
	Nutritional and diet changes and nutritional supplements (such as B vitamins and probiotics) can be useful in managing symptoms in some patients with Long COVID
	Wearable devices that track heart rate variability (HRV) are useful to guide the pacing of activity and exertion in Long COVID
	Anticoagulant and antiplatelet drugs can be used to treat a subpopulation of patients with Long COVID, as long as appropriate diagnostic tools for thrombotic endothelilitis are available and treatment is overseen by an experienced clinician
Consensus level C statements	Long COVID biomarkers are important for treatment of the disease, despite its complexity
	Pain medications can be useful for treatment of pain in Long COVID
	Nutritional and diet changes and nutritional supplements (such as vitamin B group) can be useful in treating some Long COVID patients
	Graded exercise can be useful in treating some Long COVID patients who do not have post-exertional malaise (PEM) or post-exertional symptom exacerbation (PESE)
	Vagus nerve therapies (eg cold exposure, breathwork, mindfulness, compression wear, trauma release, vagus nerve stimulators) can be useful in treating some Long COVID patients
	Pulmonary rehabilitation can be useful in treating some Long COVID patients
	Melatonin can be useful in treating some Long COVID patients
	Anticoagulant drugs can be useful in treating some Long COVID patients
	Drugs for treatment of gastroparesis and hyperacidity (e.g. proton pump inhibitors, H2-blockers) can be useful in treating Long COVID
	In treating Long COVID it is important to consider treatment of SARSCoV-2 viral persistence with treatments that have antiviral effects
	Treatments that target Vagus nerve dysfunction can be useful in managing Long COVID symptoms
	Therapies that stimulate the Vagus nerve and/or promote parasympathetic activation of the autonomic nervous system (e.g. mindfulness, breathwork, cold water exposure, cryotherapy, trauma release, cranial osteopathy, acupuncture) can be useful in treating patients with Long COVID
	Pulmonary rehabilitation (an established exercise training and education programme for people with structural lung disease) is not indicated for the majority of people with Long COVID related breathlessness
	Melatonin can be useful in treating Long COVID related insomnia
	Drugs for treatment of gastroparesis e.g. metoclopramide, domperidone, pyridostigmine, can be useful in treating Long COVID patients with dysautonomia
	Cat.: Evaluation of treatment
Consensus level A statements	Fatigue assessment tools (e.g. Epworth Sleep Scale, Fatigue Severity Scale, etc.) can be useful to measure the effects of Long COVID treatments
	For paediatric patients, back to school attendance, resuming sport, musical and other activities as normal might be a good measure of successful treatment
Consensus level B statements	The Symptom Burden Questionnaire for Long COVID (SBQ-LC) can be used to monitor the effect of treatment in patients with Long COVID
	Follow-up (repeat) examination with cognitive screening tools and physical examination can be useful to measure the effect of Long COVID treatment
	Re-imaging for specific Long COVID complications such as pulmonary embolism, myocarditis, and heart failure can be useful to monitor the response to treatment in patients with Long COVID
	If available, repeat measurement of markers of endothelial dysfunction, platelet hyperactivation and abnormal clotting physiology (such as vWF, sCD40 ligand, VEGF & micro-clot detection) can be useful to track the effect of some Long COVID treatments
	If determined safe and appropriate following detailed screening for post-exertional malaise (PEM), repeat cardiopulmonary exercise testing (CPET) can be important to monitor changes in VO2 max and anaerobic threshold and to measure the effectiveness of treatments (including rehabilitation programmes) in Long COVID
Consensus level C statements	Cognitive screening tools (e.g. Montreal Cognitive Assessment or Mini-Mental State Examination) can be useful to measure the effects of Long COVID treatments
	Repeated haemostatic tests for coagulopathy can be useful to measure the effect of Long COVID treatment
	Cat.: General research
Consensus level A statements	Research into the pathomechanism(s) of Long COVID, including relevant organ systems, is of paramount importance to long-term treatment goals
	Reducing transmission of SARS-CoV-2 will lower the incidence of Long COVID
	Viral persistence as a potential mechanism for Long COVID should be researched
	A major target area of research should be on the effects of COVID/Long COVID on the cardiac and vascular systems
	A major target area of research should be on the effects of COVID/Long COVID in children
	A major target area of research should be on the effects of COVID/Long COVID on acute and prolonged states of inflammation
	All systems in the body need to be considered in the research of Long COVID
	An international task force should be formed to develop a consensus on Long COVID research priorities and facilitate/encourage global collaborative efforts and data sharing
	A major medical/scientific research goal should be establishing pathogenesis of Long COVID
	Research should look at the future societal and economic impacts of SARS-CoV-2. This research should assess and include the potential rise in health, social and economic burdens of other chronic diseases triggered or worsened by SARS-COV-2
	A target area of research should be on the effects of COVID/Long COVID on sleep
	A major target area of research should be on the immune dysfunction associated with COVID/Long COVID
	The relationship between Long COVID and the gut microbiome/dysbiosis is an important area to research
	Mechanisms of and treatments for post-exertional malaise/post exertion symptom exacerbation (PEM/PESE) in Long COVID is an important area of research
	Investigating autonomic dysfunction in Long COVID is an important area of research
	Development of evidence-based treatment protocols for endothelial dysfunction and
	coagulopathy in COVID and Long COVID is an important area of research
	Understanding factors that exist pre-infection and during acute COVID-19 infection that predispose to development of Long COVID is an important area of research
	A target area of research should be on the effects COVID/Long COVID on mitochondrial function as well as cellular metabolism and senescence
	Markers of mitochondrial dysfunction should be investigated in Long COVID
Consensus level B statements	Research into Long COVID should assess the impact of COVID-19 on increased susceptibility to infection in the post-COVID period
	A major target area of research should be on the effects of SARSCoV-2 reinfections on COVID/Long COVID
	A major target area of research should be on the effects of COVID/Long COVID on sleep
	A major target area of research should be on the effects of COVID/Long COVID on glucose and lipid metabolism
	Cleaning indoor air is an issue that should be prioritised to lower the incidence of acute COVID-19 infections and, therefore Long COVID
	A target area of research should be on the effects of antivirals on COVID/Long COVID
	Given the link between poor oral health & adverse effects from COVID-19, a target area of research should be on the relationship between COVID/Long COVID and oral health, including the oral microbiome and periodontal disease
	Research into the pathophysiology of Long COVID should include nutritional and metabolic status, e.g. trace elements, amino acids, organic acids, intracellular minerals, trace elements, and electrolyte stores, as well as energy metabolites
Consensus level C statements	Deciding to clean indoor air is an engineering issue that should be prioritised to lower the incidence and COVID and therefore Long COVID
	A major target area of research should be on the relationship between COVID/Long COVID and oral health including the oral microbiome and periodontal disease
	Given the link between poor oral health & adverse effects from COVID-19, a target area of research should be on the relationship between COVID/Long COVID and oral health, including the oral microbiome and periodontal disease
	Cat.: Research on organ or body damage
Consensus level A statements	Damage to the nervous system might be incurred by COVID/Long COVID but does not always appear with initial symptoms
Consensus level B statements	Damage to the liver, pancreas, kidneys, and/or skin might occur in COVID/Long COVID but may not always appear with initial symptoms or within the first few months
	Damage to the patient’s endothelium and/or microvasculature might occur in COVID/Long COVID but does not always appear with initial symptoms
	Covid-19 may cause direct damage to cardiomyocytes. This may occur in the absence of a rise in cardiac biomarkers such as troponin
	Covid-19 may be associated with a higher risk of dementia or acceleration of dementia
	Multi-organ damage affecting the liver, pancreas, kidneys, and/or skin can occur in COVID/Long COVID but may not always appear with initial symptoms or within the first few months
Consensus level C statements	Clinical research has already demonstrated that COVID-19 infection can trigger or accelerate neurodegenerative diseases like dementia, Parkinson’s disease, and motor-neuron diseases
	Cat.: Research on children and young people
Consensus level A statements	The impact of Long COVID on children's attendance of and performance in school should be researched
	The impact of Long COVID on new onset diabetes in children should be researched
	The impact of Long COVID on the immune systems of children should be researched
	The long-term impact of Long COVID on children should be researched
	There is a need to study the impact of repeated COVID-19 infections on Long COVID in both children and adults
	Research into the physiological effects of Long COVID in children, including thrombotic endotheliitis (e.g. endothelial damage, activated platelets and micro-clots), viral persistence, and gastrointestinal impacts should be a priority
	The impact of repeated SARS-CoV2 infections on children’s behavior, cognition, concentration, and mental health should be an area of research priority
	A research priority should be to investigate why some children develop paediatric acute-onset neuropsychiatric syndrome (PANS) or MIS-C after acute COVID-19 infection and others do not
	Investigating the impact of COVID-19 vaccinations and boosters on the incidence and severity of Long COVID in children who have had COVID-19 infection should be a priority
Consensus level B statements	The impact of Long COVID on the development of narcolepsy and sleep disordered breathing in children should be researched
	Exploring a comprehensive approach to the treatment of Long COVID in children, encompassing biological, psychological, social, and ecological factors should be a priority
	Cat.: Long COVID and vaccination
Consensus level A statements	Where vaccination has led to or impacted Long COVID symptoms this should be carefully researched and patient risks from different vaccines identified to inform guidelines
	Where vaccination has led to vaccine injury or impacted Long COVID symptoms this should be treated
Consensus level B statements	Long COVID-like symptoms can occur following vaccination
	Vaccination can cause ongoing symptoms in some people, and this should be researched
	Vaccination can reduce the risk of Long COVID but does not prevent it
	An area for research should be which vaccines are least likely to worsen symptoms in patients with pre-existing Long COVID
	Research needs to look at which Long COVID patients may be at increased risk of adverse effects following COVID vaccination (such as those with ME/CFS) so that individualised tailored decisions can be made
Consensus level C statements	SARS-CoV-2 vaccination can trigger a syndrome similar to Long COVID in some individuals
	Cat.: Funding, economic and societal issues
Consensus level A statements	All funding towards Long COVID research should be funded in a transparent manner
	A major target for research should be the economic and societal impacts of Long COVID
	Funding should be allocated to research Long COVID AND its impact on society
	Schools should be required to offer remote learning and other educational aids for children with Long COVID
Consensus level B statements	Health insurance companies should support research into Long COVID and assume a more active role in the solution
	Health policies that encourage children to attend school while actively infected with COVID are likely to further increase absences rather than aid attendance rates and may result in increased Long COVID in Children
	Health policies that encourage children to attend school while actively infected with COVID are likely to increase Long COVID in parents
Consensus level C statements	Corporate entities have a responsibility to contribute to the funding of Long COVID research

The strongest consensus areas were in diagnosis and clinical assessment (1 statement unanimous, 19 with A-level, 13 with B-level and 3 with C-level statements), general research (24 A-level statements, 13 B-level and 6 C-level), research on children and young people (15 A-level statements and 6 B-level), and funding, economic and societal issues (4 A-level statements, 3 B-level, and 1 C level). Organ or body damage (1 A-level, 8 B-level and 1 C-level statements) and Long COVID and vaccines (2 A-level, 7 B-level, and 1 C-level statements) require more research before we can reach a broad consensus. Not surprisingly, there less congruence on treatment (9 A-level, 13 B-level and 15 C-level statements) and evaluation of treatment (2 A-level, 5 B-level and 2 C-level statements) as clinicians around the world have adopted their own practices in the absence of clear guidelines or a strong evidence-base. This reinforces the need for translational research and trials.

Statements not reaching consensus are listed in Table S2 of the supporting information. Of these 34 statements, 8 deal with definition, 5 with diagnosis, 17 with treatment, 2 with evaluation of treatment, 1 statement on vaccination improving Long COVID and 1 statement nearly reaching consensus calling for routine cognitive impairment testing in critical professions (discussed below). It is not surprising that definition and treatment have the most statements not reaching consensus as they are the areas most in need of clarity in understanding Long COVID.

## Discussion

### Long COVID definition

The WHO [12], CDC [13], USA NESAM [14] and others have proposed candidate definitions (Table 2), but there is currently no single unified definition of Long COVID, which is a detriment to the research, diagnostics, treatment, and patient rights. The consensus agreement emphasises that functional impairment, reduced effort tolerance, new-onset or worsening of pre-existing conditions, abnormalities in clinical parameters or medical imaging, and other detectable systemic pathology should be included in the definition or as a distinct clinical category, regardless of the presence or absence of associated symptoms. Of the listed definitions, this component is currently present only in the USA NESAM definition, which includes not only symptoms but also diagnosable conditions which may or may not produce recognizable symptoms. These include interstitial lung disease and hypoxemia, cardiovascular disease and arrhythmias, cognitive impairment, stroke, hyperlipidemia, blood clots, chronic kidney disease, and many other diseases which are known to often remain clinically silent until late in their natural history when the physiological reserve is exhausted. The main contributions of the work presented here are recognising (1) the role of functional impairment in Long COVID; and (2) the diversity of distinct associated conditions and different treatments.

**Table 2:** Definitions of Long COVID by WHO, CDC, USA NAS

WHO Definition	A condition that occurs in individuals with a history of probable or confirmed SARS-CoV-2 infection, usually three months from the onset of COVID-19, with symptoms that last for at least two months and cannot be explained by alternative diagnosis. Common symptoms include fatigue, shortness of breath, cognitive dysfunction, and others witch generally impact everyday functioning
CDC Definition	A chronic condition that occurs after SARS-CoV-2 infection and is present for at least 3 months. Long COVID includes a wide range of symptoms or conditions that may improve, worsen, or be ongoing
USA NAM Definition	An infection associated chronic condition that occurs after SARS-CoV-2 infection and is present for at least 3 months as a continuous, relapsing and remitting, or progressive disease state that affects one or more organ systems. A complete enumeration of possible signs, symptoms and diagnosable conditions of LC would have hundreds of entries. Any organ system can be involved, with single or multiple symptoms or single or multiple diagnosable conditions

### Diagnosis recommendations

There was unanimous consensus for a need to educate all health care workers about the possible complications following SARS-CoV-2 infection. The panel was clear that patients should be listened to, appropriately investigated, and supported.

Long COVID is multi-systemic and may present with different phenotypes that fall under the umbrella of Long COVID. Thus, the process of diagnosis must reflect the complexity of the condition, and the variability of signs and symptoms among individuals may present a special challenge for clinicians. The first step in the diagnostic process is the determination that the individual patient has experienced a prolonged change in health status and a decline in functioning following an acute SARS-CoV-2 infection.

This consensus underlines the need for a nuanced and multi-dimensional diagnostic approach, considering the broad spectrum of Long COVID manifestations. The necessity for individualised diagnostic strategies to effectively capture and manage the disease's complexity is also emphasized. A summary of significant diagnostic criteria derived from the consensus statements is given in Table 3.

**Table 3:**
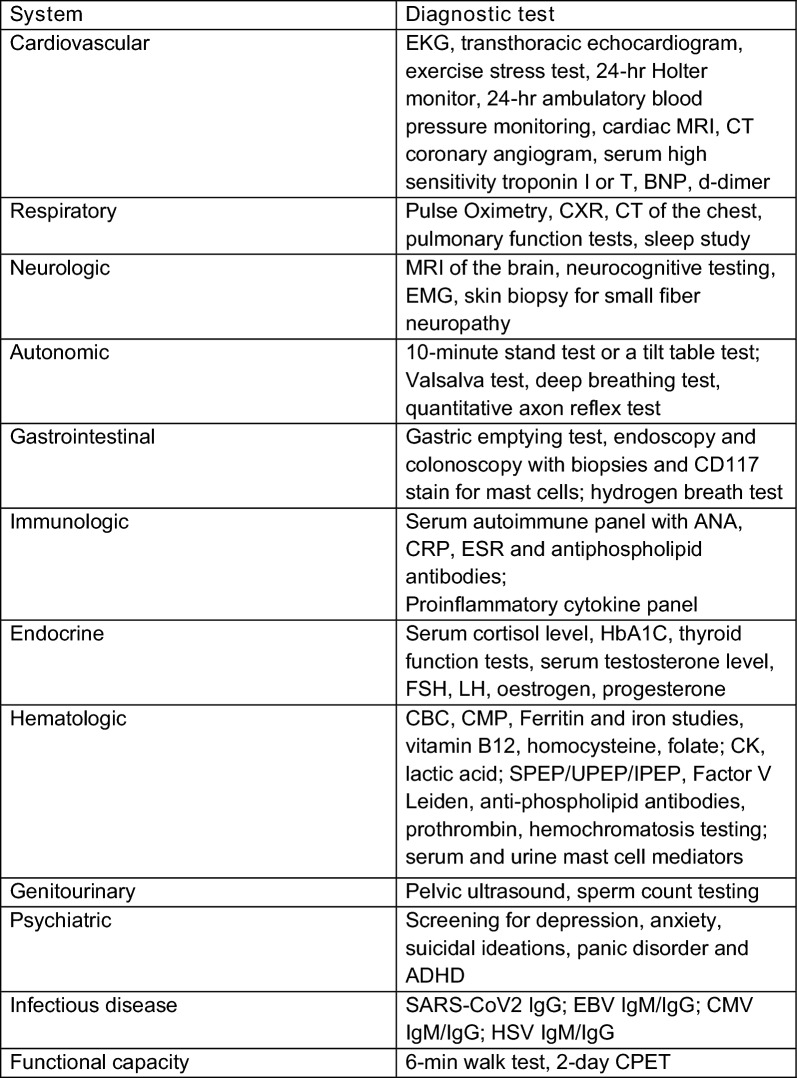
Recommended diagnostic tests available to clinicians for evaluation of patients with Long COVID based on clinical history and examination

The change in health status may include not only the appearance of symptoms and signs of illness, but decreased ability to perform or greater effort needed to maintain activities of daily living compared to the pre-COVID-19 state. Self-reported impairment of energy level, cognitive function, changes in mood, sleep or social interactions and exercise tolerance are all important indicators. The second diagnostic step is typically an assessment of possible alterations in cardiovascular, respiratory, metabolic, endocrine, renal or neurological health, as each of these systems alone or in combination may be affected in patients with Long COVID.

Currently, there are no evidence-based guidelines on the diagnosis of Long COVID and no specific validated diagnostic tests that are clinically available to diagnose Long COVID. Thus, the diagnosis is based primarily on clinical assessment consisting of detailed history and physical exam, in conjunction with the currently available diagnostic tests that help identify objective evidence of possible underlying pathophysiology and possible post-COVID-19 conditions. Several consensus guidance statements on the assessment of Long COVID and post-COVID-19 conditions have been developed to help guide healthcare practitioners in the diagnostic approach to Long COVID [15–17].

Clearly, there is a great need to develop a spectrum of validated, clinically useful and easily accessible diagnostic tests for patients with Long COVID. These may assess endothelial, microclotting, as well as mitochondrial functions, oral and gut microbiome, and inflammatory, immunologic, autoimmune and hypercoagulable state biomarkers. For example, a number of reports have discussed gut microbiome dysbiosis resulting from COVID infection, and affecting other organs [18–20]. Biomarkers may include serum cytokines and antibodies that underlie the pathophysiology of Long COVID. Several markers have been suggested as potential diagnostic biomarkers for Long COVID, including Interleukin 6 [21], C-reactive protein, tumour necrosis factor alpha, neurofilament light chain, glial fibrillary acidic protein and transforming growth factor beta [22–24]. Aside from C-reactive protein and other inflammatory and autoimmune markers available as part of the standard diagnostic serum panels, most of these biomarkers are not readily available or easily accessible in clinical practice around the world. Bridging the gap between testing available to researchers and those available to clinicians is paramount to improving diagnostic capabilities and investigations available to patients and their treating physicians.

## Treatment and evaluation of treatment

To-date, like diagnostic approaches, there are no evidence-based guidelines on the treatment of Long COVID and no specific validated procedures that are clinically available to treat this complex disorder. Consensus shows it requires an individual and tailored approach to each patient accounting for patient history and physical examination and that multidisciplinary team management is beneficial.

Treatment has focused predominantly on the demonstrable pathologies and manifestations of their many varied presentations and identified processes. Midodrine, ivabradine beta-blockers, and fludrocortisone can be used for POTS [25–27], whereas a combination of 5-HT1 inhibitors and two antihistamines can be helpful for management of symptoms of MCAS [28]. Statins can be used for hyperlipidaemia and melatonin for sleep dysrhythmia and endothelial protection. There have been several randomised controlled studies but no single unifying therapy. Considerable gaps remain in treatment protocols including the use of anticoagulants, probiotics [29], and mitochondrial supplements. Larger trials of antivirals for both prevention and treatment of Long COVID are needed. A small RCT showed no benefit of 15 days of nirmatrelvir-ritonavir [30]. Whilst there is a vast array of therapies for specific issues, there are few well-performed RCTs for treatment efficacy in Long COVID.

A summary of significant treatment criteria from consensus is given in the Supporting Information (Table S3). Clearly there is agreement that multidisciplinary team tailored therapies are essential [31]. The management of POTS, sleep disturbance and MCAS were highly conserved across both Delphi rounds 2 and 3. Attention to clotting abnormalities, whilst considered important, the consensus suggests that this is best undertaken when biomarkers are available, and expertise is required for treatment oversight.

There was excellent consensus for the appropriate treatment of mood disorders and the conjunctive value of psychological therapies and support [32]. Formal neurocognitive evaluation both at presentation and at follow-up is strongly supported. The usual standard evaluation tools were considered inadequate and more comprehensive testing was strongly recommended. This is critical as COVID-19 can lead to long-term cognitive impairment [33].

There was considerable caution around exercise and pulmonary rehabilitation, particularly in the absence of an adequate clinical evaluation and exclusion of POTS and PEMS [34]. The value of non-invasive vagus nerve stimulation therapies (including stimulators, cold exposure breathwork, mindfulness, and trauma release) was supported, but not strongly. This may reflect local practices and experience as well as the lack of RCT’s for external stimulators and the efficacy of non-conventional treatments. Tracking devices for assessment of heart rate, sleep quality and pacing for patients, whilst Level B, may have value for both clinicians and patients. Group data for prospective evaluation of therapeutics may be where this lies in the future.

## Future research focus

The overarching consensus from this Delphi, with regards to research, is the need for a designed framework. This will allow for a structured approach to addressing Long COVID, outlining key focus areas of interest, concern, and need.

The consensus statements on research and particularly on organ damage, align closely with the existing literature, which increasingly supports a multi-system involvement in Long COVID [6, 35]. Research to date indicates a complex interplay of factors that may contribute to the persistence of symptoms, suggesting the need for a holistic approach in future studies. Ongoing research has shown that Long COVID impacts the immune system [36] and there is an increased risk of cardiovascular complications such as heart attacks, coronary heart disease, heart failure, and deep vein thrombosis among those infected [37–39]. These cardiovascular issues are often precipitated by disruptions in endothelial cell function, which regulate the flow of substances into and out of tissues and are a focal point of the virus’s deleterious effects [40]. The virus also poses significant neurological risks [41].

Consistent with the consensus agreement, persistent symptoms have been associated with metabolic and endocrine systems, potentially leading to diabetes through mechanisms involving pancreatic dysfunction and altered insulin responses [42]. Reproductive health issues in both males and females have been noted [43–45]; with the virus affecting hormonal balances, potentially disrupting menstrual cycles, impairing fertility, and complicating pregnancies. Kidney damage from the virus can escalate to chronic kidney disease, and gastrointestinal symptoms, may persist or lead to chronic conditions, reflecting the virus's ability to infect intestinal cells and possibly alter the gut microbiome [46, 47]. This extensive involvement of diverse bodily systems illustrates the critical need for a multi-disciplinary approach in research and treatment strategies for COVID-19 and Long COVID, highlighting the virus's ability to cause systemic damage beyond the respiratory system.

The research directions highlighted in the consensus emphasise the importance of investigating the relationship between Long COVID and other health conditions, such as sleep disorders, dysbiosis, and PEM. This suggests a complex interplay between Long COVID and pre-existing vulnerabilities or concurrent health issues. As most of the COVID-19-induced pathophysiology is immune-mediated or even immunologically driven, interactions with any existing or future inflammatory conditions are likely to be important at an individual level. The emphasis on endothelial dysfunction, coagulopathy, and mitochondrial dysfunction points to a nuanced understanding of the disease's pathophysiology, indicating potential pathways for targeted treatments.

Global SARS-CoV2 vaccination programs have saved lives, and research shows on a population basis that COVID vaccines reduce the risk of long COVID [48, 49]. However less is known about the individual effects of vaccination on patients who have pre-existing immune mediated inflammation from Long Covid. A common question our long COVID clinicians are asked by patients is whether the vaccine will make their long covid symptoms better or worse. To our knowledge this has not been studied and our experts showed consensus agreement that this was an important area for further research so clinicians can give patients scientifically informed answers. There was not agreement with the statement, ‘Long COVID can improve with vaccination.’ The consensus and nonconsensus show that vaccination and Long COVID is an issue that is still not clear.

In parallel, the consensus points to the need to address factors that may lower the incidence of Long COVID, such as reducing SARS-CoV-2 transmission and improving indoor air quality, highlighting the intersection between public health measures and individual health outcomes. This underscores the complexity of Long COVID and the necessity for longitudinal studies to fully comprehend its long-term implications.

The growing number of reports and studies on Long COVID in children show that dedicated research into how the condition affects this vulnerable population is imperative. Consensus points from recent rounds highlight the importance of studying the effects on educational performance, mental health, and physiological development. These priorities resonate with recent findings which underscore the unique challenges faced by children with Long COVID [50], including the potential for significant developmental disruptions and the need for tailored clinical approaches.

Existing evidence on Long COVID in children is scarce, partly due to a lack of a standardised case definition, short follow up duration, and heterogenous study designs, resulting in wide variation of reported outcomes [51]. Stigmatisation of children, due to a lack of an understanding or ignorance about the disease in children has also been reported [45]. There has, however, been evidence for widespread endothelial damage in children with Long COVID [52, 53].

A pivotal area of concern is the interaction between Long COVID and the education system, highlighting the need to explore how the condition affects school attendance and performance. This extends to the potential cognitive repercussions, including impacts on the developing brain and resultant challenges in learning and development. Of note, meta-analysis identified five paediatric studies documenting abnormal brain imaging findings in children and young people with Long COVID [54]. Moreover, the physical health consequences of Long COVID in children, such as new onset diabetes and alterations in the immune system, underscore the critical need for studies tailored to the paediatric population. The investigation into the long-term physiological effects, including those as specific as thrombotic endotheliitis, presents a clear directive for future research endeavours. This is particularly relevant considering recent pilot studies documenting objective physiological abnormalities in young patients with Long COVID, including dysautonomia [55, 56], pathological cardiopulmonary exercise testing [57], immune dysregulation [58], and platelet activation [53].

It is important to note that the burden of post-acute sequelae of COVID-19 may go beyond what is currently referred to as Long COVID. For example, there may be sub-clinical impacts following a SARS-CoV-2 infection as well as symptoms that may not be considered related to Long COVID. Unrecognised sequelae include new onset health conditions [3, 59] or worsening of pre-existing health conditions [60] in adults and children. Diverse medical presentations may not be considered Long COVID due to a lack of awareness. An area of research agreed on by consensus is to develop biomarkers for Long COVID, and this can be extended to unrecognised conditions including the heart, brain, vasculature, and more.

## Long COVID funding, economic impact and societal issues

The consensus reached through the modified Delphi process offers a nuanced view of the global response required to address the socioeconomic challenges of Long COVID. More effective strategies and interventions are needed, especially for more impacted groups, since disparities appear exacerbated by Long COVID. A mixed-methods study across five countries explored the economic and social impacts of COVID-19 revealing significant disparities based on age, education, household size, and income [61]. Also, the well-known COVIDENCE UK study highlights the economic vulnerability caused by COVID-19, showing increased short-term household income inadequacy and long-term sickness absence from work, indicating a cycle of impaired health and poor economic outcomes [62]. Additionally, Long COVID requires a reinforced epidemiological surveillance program or periodic reviews for the health personnel who fall ill with it, having been the ones who provided direct care to patients who suffered from COVID-19.

Studies have also quantified impacts of Long COVID on workforce participation, sick leave, disability, and economic activity. A UK cohort study quantified the health and economic burden of Long COVID, finding substantial impacts on health-related quality-of-life and emphasising the need for continued support and research for those affected [63]. Another population-based cohort study in Hong Kong evaluated the long-term spill-over effects of COVID-19 on people with non-communicable diseases, showing significant disruptions in health outcomes and healthcare costs, stressing the need for optimised care [64].

In synthesising the consensus points, it becomes evident that tackling Long COVID requires a concerted effort from all sectors of society. For example, Uwishema et al. (2022) highlight how COVID-19 disrupts healthcare access for neurological patients in Africa, a challenge likely magnified by Long COVID’s chronic burden [65] and Uwishema and Boon emphasize addressing neurological care inequities, a priority echoed in Long COVID’s global burden on underserved populations [66]. The insights gained from this modified Delphi study not only inform immediate policy and research priorities but also illustrate the broader societal shifts necessary to deal with the pandemic’s long-term effects effectively. The consensus conclusions can be aggregated into the following overarching considerations: (1) an interdisciplinary approach is needed to address the issue of Long COVID; (2) educational adjustments and policy implications need to be discussed and implemented; and (3) corporate responsibility and public health funding are needed. Additionally, the near consensus calling for routine cognitive impairment testing in critical professions underscores the practical implications of Long COVID on workforce safety and productivity.

## Strengths and limitations

One of the strengths of this study is the broad scope covering the entire issue of Long COVID. Another strength is we minimized potential bias by generating a large, geographically and diverse panel from multiple sources where nominees from the working group were combined with a comprehensive literature search to identify Long COVID authors. These made up most of the panellists. Additionally, we implemented a modified Delphi methodology where we used experts identified by literature search to answer questions about the most important topics in Long COVID. This allowed the most unbiased approach possible to an understanding of this complex topic and development of subsequent statements. The large sampling of physicians working with LC patients, and researchers, gives this consensus credibility, especially in the areas of understanding what Long COVID looks like, how it is diagnosed and treated, as well as what should be researched. Finally, statements from the second round of Delphi process were made clearer for round three with the help of open-ended comments after each section of the survey.

The Delphi method has many advantages, but limitations also exist. One limitation is that the survey was voluntary and, hence, this self-selection might have omitted some experts. However, everyone who wanted to participate was able to do so. It is important to note that a small number of the panel experts (16%) were also on the extended committee. This number is small making it unlikely to be significant. Another limitation is that Long COVID is an evolving syndrome, and the science reviewed in this paper covers a time period from mid-2023 to mid-2024; the scientific evidence will have to be revisited later. A follow up survey might be useful when diagnosis and treatment are more refined. One consideration that is difficult to address is that there might be insufficient data due to lack of good healthcare protocols especially in lower-middle income countries and issues related to stigmatization with people not going to the hospital, missed data, lack of funding and people not able to afford medical visits and therefore not being assessed for Long COVID. Furthermore, differences in how long covid cases are managed in high-income vs low-middle income countries might lead to differences across panelists. This limitation should be addressed by the broad range of countries used in this survey.

An important concern can be raised that there is a lack of randomised controlled trials in the areas of diagnosis and treatment of Long COVID. These still need to be developed, but our consensus establishes the groundwork for implementation of clinical care for people with Long COVID.

## Conclusions

This modified Delphi study is the first to provide international consensus regarding the clinical evaluation and medical investigation of Long COVID with expert consensus recommendations to physicians. Gaining consensus agreement from 179 experts around the globe we establish conditions for diagnosis of different subgroups within the Long COVID umbrella. Strong consensus was gained for assessment and treatment of Long COVID-associated conditions, including POTS, MCAS, insomnia, new onset dyslipidaemia, diabetes, and hypertension. Consensus was also achieved that cardio-metabolic disturbance should be ruled out before prescribing graded exercise therapy as treatment. Biomarkers, where available, may be useful when monitoring treatment response to Long COVID.

Our expert panel agreed that further research was urgently needed for Long COVID. It was recommended that an international task force should be developed to oversee research priorities and facilitate/encourage global collaborative efforts and data sharing. Instead of abandoning public health related to infectious diseases, governments need to reaffirm priorities. There are over 400 million people worldwide affected by Long COVID and it is not just for covid, but for all post viral syndromes, that this work needs to be done. Clear consensus was reached that the impacts of COVID-19 infection on children should be a research priority (e.g. prevention of transmission in schools, long-term impacts of infections, impacts on learning/development, etc.). Consensus was also reached on the need to determine the effects of Long COVID on societies and economies, and that governments need to prioritise investment in public health protections to prevent reinfections.

## Supplementary Information 

Additional file 1.

## Data Availability

No datasets were generated or analysed during the current study.
