# Correction: Long COVID clinical evaluation, research and impact on society: a global expert consensus

**DOI:** 10.1186/s12941-025-00803-w

**Published:** 2025-07-10

**Authors:** Andrew G. Ewing, David Joffe, Svetlana Blitshteyn, Anna E. S. Brooks, Julien Wist, Yaneer Bar-Yam, Stephane Bilodeau, Jennifer Curtin, Rae Duncan, Mark Faghy, Leo Galland, Etheresia Pretorius, Spela Salamon, Danilo Buonsenso, Claire Hastie, Binita Kane, M. Asad Khan, Amos Lal, Dennis Lau, Raina MacIntyre, Sammie McFarland, Daniel Munblit, Jeremy Nicholson, Hanna M. Ollila, David Putrino, Alberto Rosario, Timothy Tan, The Long COVID consensus expert panel (Table S1), Lawrence B. Afrin, Carlos Arturo Alvarez Moreno, Nisreen Alwan, Sampath Kumar Amaravadi, Harashen Ashraf, Veronica Athie Morales, Philip Atkinson, Robert Axtell, Cipatli Ayuzo, Alba Azola, James Bagley, Sergio Bagnato, Amitava Banerjee, Yaneer Bar Yam, John Barry, Daniela Bassi-Dibai, Anne Bheréur, Stephane Bilodeau, Gregory Bix, Jose-Ramon Blanco, Svetlana Blitshteyn, Hector Bonilla, Angela Bowers, Anna E. S. Brooks, Marie Bruyneel, Alexandra Brugler Yonts, Danilo Buonsenso, Gunnar Bücker, Kathleen Capaccione, Harriet Carroll, Pascal Cathebras, Ashish Chaudhry, Bela Cheda, Karin Chia, Pratima Chowdary, Tae Hwan Chung, Leslie Conner, Caterina Conte, Jennifer Curtin, Caroline Dalton, Kara Darling, Helen Davies, Kate Davies, David Davies-Payne, Kevin Deans, Luis Del Carpio Orantes, Anne Doherty, Theresa Dowell, Rae Duncan, Raechel A. Evans, Andrew Ewing, Mark Faghy, Emilia Liana Falcone, Bingwen Eugene Fan, Emily Fatakhov, Kelly Fearnley, Artur Fedorowski, Carlo Ferrarese, Francesco Ferraro, Gemma Figtree, Laurie Findlay, Alberto Fortini, Matteo Foschi, Francois Gagnon, Nick Gall, Leo Galland, Natasha Gerbis, Ali Gholamrezanezhad, Amine Ghram, Andréa Lúcia Goncalves da Silva, Helen Goss, Stephanie Grach, Caroline Gregoire, Gary Groot, Riccardo Guanà, Eric Guedj, Claire Hastie, Merel Hellemons, Melanie Hoppers, Mohammad-Salar Hosseini, Lee Ingle, Linn Jarte, David Joffe, Binita Kane, Leslie Kasza, David Kaufman, Sundeep Kaul, Douglas Kell, Daphne Kemp, Robin Kerr, Asad Khan, Abbas Khushnood, SangYun Kim, Cheryl Koh, Nik Kudiersky, Katharina Kurz, Gonzalo Labarca, Amos Lal, Francisco Landi, Dennis Lau, Jaco Laubscher, Joshua Leisk, Jacob Leone, Graham Lloyd-Jones, C. Raina MacIntyre, Kushal Madan, Stuart Malcolm, Harsha Master, Sammie McFarland, Gez Medinger, Yolanda Meije, Alexios-Fotios Mentis, Francisco Mera-Cordero, David Miller, Daniel Munblit, Bethan Myers, Hiten Naik, Javaid Nauman, Jeremy Nicholson, Josef Niebauer, Estefania Nova-Lamperti, Maria Olivier, Otieno Martin Ong’wen, Rebecca Owen, Kelly O’Brien, Stefano Pallanti, Roger Paredes, Tina Peers, Alice Perlowski, Colin Pidgeon, Julianne Pitzele, Chris Ponting, Anna Porter, Etheresia Pretorius, Amy Proal, Milo Puhan, David Putrino, Shaun Peter Qureshi, Sunil Raina, Nandini Raj, Ashwin Rajenesh, Clare Rayner, Alfonso J. Rodriguez-Morales, Luca Roncati, Alberto Rosario, Cilla Rosen, Ilene Ruhoy, Leslie Rydberg, Špela Šalamon, Carmen Scheibenbogen, Sarah Schenck, Katharine Seagly, Jaffer Shah, Ben Sinclair, Ioakim Spyridopoulos, Warren Tate, Claire Taylor, Catherine Tewolde, Alain Thierry, Callum Thomas, Tracey Thompson, Irene Tosetti, Eleonora Trecca, Jordan Vaughn, Chantelle Venter, Amber Vermeesch, Joe Vipond, Guiulia Vivaldi, Maxine Waters, William Weir, Emma Weisblatt, Julien Wist, May Wong, Rob Wüst, Azfar Zaman, James Zhang

**Affiliations:** 1 Department of Chemistry and Molecular Biology, University of Gothenburg, Gothenburg, Sweden; 2 World Health Network Long Covid Expert Advisory Group, Cambridge, USA MA; 3 Respiratory and Sleep Medicine, Royal North Shore Hospital, St Leonards, Australia; 4 Woolcock Institute of Medical Research (Sleep Group), Sydney, Australia; 5 Department of Neurology, University at Buffalo Jacobs School of Medicine, Buffalo, NY USA; 6 Dysautonomia Clinic, Williamsville, NY USA; 7 Liggins Institute, The University of Auckland, Auckland, New Zealand; 8 School of Biological Sciences, Faculty of Science, The University of Auckland, Auckland, New Zealand; 9 The Maurice Wilkins Centre, Auckland, New Zealand; 10 Australian National Phenome Centre, Murdoch University, Murdoch, Australia; 11 Imperial College London, London, UK; 12 Chemistry Department, Universidad del Valle, Cali, Colombia; 13 New England Complex Systems Institute, Cambridge, MA USA; 14 Department of Bioengineering, McGill University, Montreal, Canada; 15 Real Time Health Monitoring, San Francisco, CA USA; 16 The Newcastle Hospitals NHS Foundation Trust, Newcastle Upon Tyne, UK; 17 Biomedical and Clinical Exercise Science Research Theme, University of Derby, Derby, UK; 18 Foundation for Integrated Medicine, New York, NY USA; 19 Department of Physiological Sciences, Faculty of Science, Stellenbosch University, Stellenbosch, Western Cape South Africa; 20 Department of Biochemistry, Cell and Systems Biology, Institute of Systems, Molecular and Integrative Biology, Faculty of Health and Life Sciences, University of Liverpool, Liverpool, UK; 21 Department of Woman and Child Health and Public Health, Fondazione Policlinico Universitario A. Gemelli IRCCS, Rome, Italy; 22 Founding Member, Long Covid Support, London, UK; 23 Manchester University Foundation Trust, School for Biological Sciences, University of Manchester, Manchester, UK; 24 Directorate of Respiratory Medicine, Manchester University Hospitals, North West Lung Centre, Manchester, M23 9LT UK; 25 Division of Pulmonary, Critical Care and Sleep Medicine, Mayo Clinic, Rochester, MN USA; 26 The University of Adelaide and Royal Adelaide Hospital, Adelaide, SA Australia; 27 Biosecurity Program, Kirby Institute, University of New South Wales, Sydney, Australia; 28 CEO & Founder Long Covid Kids, London, UK; 29 Care for Long Term Conditions Division, Florence Nightingale Faculty of Nursing, Midwifery and Palliative Care, King’s College London, London, UK; 30 Australian National Phenome Centre, Murdoch University, Perth, WA Australia; 31 Faculty of Health and Medical Sciences, University of Western Australia, Crawley, Australia; 32 Imperial College London, London, UK; 33 Nanyang Technological University, Singapore, Singapore; 34 Regional Adjunct Professor, Khon Kaen University, Khon Kaen, Thailand; 35 Institute for Molecular Medicine Finland, FIMM, HiLIFE, University of Helsinki, Helsinki, Finland; 36 Broad Institute of Harvard and MIT and Center of Genomic Medicine, Massachusetts General Hospital, Boston, MA USA; 37 Department of Anesthesia, Critical Care and Pain Medicine, Massachusetts General Hospital, Boston, MA USA; 38 Centre for Genomic Medicine, Massachusetts General Hospital, Harvard Medical School, Boston, MA USA; 39 Cohen Center for Recovery From Complex Chronic Illness, Department of Rehabilitation and Human Performance, Icahn School of Medicine at Mount Sinai, New York, NY USA; 40 Infection Prevention Team, World Health Network, Cambridge, MA USA; 41 Consultant Cardiologist, Westmead and Blacktown Hospitals, Sydney, Australia; 42 Conjoint Professor, School of Medicine, Western Sydney University, Sydney, Australia; 43 Conjoint Clinical Associate Professor Sydney Medical School, Sydney University, Sydney, Australia; 44 Adjunct Associate Professor, School of Medical Sciences, Faculty of Medicine, University of New South Wales, Sydney, Australia; 45 Area Pediatrica, Dipartimento di Scienza Della Vita e Sanità Pubblica, Università Cattolica del Sacro Cuore, Rome, Italy; 46 Department of Paediatrics and Paediatric Infectious Diseases, Institute of Child’s Health, Sechenov First Moscow State Medical University (Sechenov University), Moscow, Russia

**Correction: Ann Clin Microbiol Antimicrob (2025) 24:27** 10.1186/s12941-025-00793-9

In the original publication of this article [1], the collaborator names of The Long COVID consensus expert panel (Table S1) were not tagged in the XML metadata and the affiliations of the author Daniel Munblit were incomplete. These errors have been updated with this correction article. The original article has been updated.

The collaborator names are tagged in the XML metadata and listed under Acknowledgement section.

**Acknowledgements**

We thank Sharon Fitzgerald of the World Health Network for the tremendous job of handling communications, setting up the surveys, receiving results and assembling them into excel files.

The Long COVID consensus expert panel–List of expert panel list authors and affiliations:

Lawrence B. Afrin—AIM Center for Personalized Medicine, Purchase, New York, USA; Carlos Arturo Alvarez Moreno—Universidad Nacional de Colombia; Clinica Colsanitas, Colombia; Nisreen Alwan—University of Southampton, UK; Sampath Kumar Amaravadi—School of Sport, Exercise and Rehabilitation Sciences, University of Birmingham, Birmingham, UK; Harashen Ashraf—Division of Cardiology, Children’s National Heart Center, Children’s National Hospital, Department of Pediatrics, The George Washington University School of Medicine & Health Sciences, Washington, DC, USA; Veronica Athie Morales—ConCiencia ECAI, Atizapan de Zaragoze, Mexico; Philip Atkinson—Consultant Occupational Physician, University of Morecambe Bay NHS Trust, UK; Robert Axtell—Southern Connecticut State University, Health & Movement Sciences, CT, USA; Cipatli Ayuzo—Tecnologico de Monterrey, NL Mexico; Alba Azola—Johns Hopkins School of Medicine, Department of Physical Medicine and Rehabilitation, Baltimore, USA; James Bagley—Department of Kinesiology, San Francisco State University, San Francisco, CA, USA; Sergio Bagnato—Unit of Neurophysiology and Unit for Severe Acquired Brain Injuries, Giuseppe Giglio Foundation, Cefalù, Italy; Amitava Banerjee—Institute of Health Informatics, University College London, London, UK; Yaneer Yam* Bar—Yaneer World Health Network, New England Complex Systems Institute, Cambridge, MA; John Barry—Good Shepherd Penn Partners University City, Philadelphia, USA; Daniela Bassi-Dibai—Universidade Cuema y Centro Universitário Santa Terezinha CEST, São Luís, MA, Brazil; Anne Bheréur—Universite de Montreal, Montreal, Quebec, Canada; Stephane Bilodeau*—World Health Network and Department of Bioengineering, McGill University, Montreal, Canada; Gregory Bix—Tulane University School of Medicine, Clinical Neuroscience Research Center, New Orleans, LA, USA; Jose-Ramon Blanco—Hospital Universitario San Pedro – CIBIR, La Rioja, Spain; Svetlana Blitshteyn*—Department of Neurology, University at Buffalo Jacobs School of Medicine, Buffalo, NY; Dysautonomia Clinic, Williamsville, NY, USA; Hector Bonilla—Stanford Post-Acute COVID-19 Syndrome Clinic, Palo Alto, CA, USA; Angela Bowers—Southlake Dermatology, Southlake, TX, USA; Anna E.S. Brooks*—Liggins Institute, The University of Auckland, Auckland, New Zealand; School of Biological Sciences, Faculty of Science, The University of Auckland, Auckland, New Zealand; The Maurice Wilkins Centre, Auckland, New Zealand; Marie Bruyneel—Department of Pneumology, CHU Saint-Pierre, Brussels, Belgium and Université Libre de, Bruxelles, Brussels, Belgium; Department of Pneumology, CHU Brugmann, Brussels, Belgium and Université Libre de Bruxelles, Brussels, Belgium; Alexandra Brugler Yonts—Children’s National Hospital/ The George Washington University School of Medicine and Health Sciences in Washington, DC, USA; Danilo Buonsenso*—Department of Woman and Child Health and Public Health, Fondazione Policlinico Universitario A. Gemelli IRCCS, Rome, Italy; Gunnar Bücker—Nephrologische Praxis in Osnabrück, Germany; Kathleen Capaccione—Department of Radiology, Columbia University, New York, NY, USA; Harriet Carroll—Long Covid Scientific Consultancy, UK; University of Aberdeen, UK; Lund University, Sweden; Pascal Cathebras—Department of internal medicine, University Hospital & Jean-Monnet University, Saint-Etienne, France; Ashish Chaudhry—KD Haemophilia and Thrombosis Centre, Royal Free Hospital, Univ. College London, UK; Bela Cheda—Center for Complex Diseases, Mountain View, CA, USA; Karin Chia—North Shore Private Hospital, St. Leonards, NSW, Australia; Pratima Chowdary—Univ. College London; KD Comprehensive Care Haemophlia Centre; Haemophlia, Rheumatology and Haematology Royal Free London NHS Foundation Trust, UK; Tae Hwan Chung—Johns Hopkins Postural Orthostatic Tachycardia Syndrome (POTS) Program, Lutherville, MD, USA; Leslie Conner—Real Time Health Monitoring, San Francisco, CA, USA; Caterina Conte—IRCCS MultiMedica, Milan, Italy; Università San Raffaele, Rome, Italy; Jennifer Curtin*—Real Time Health Monitoring, San Francisco, CA, USA; Caroline Dalton—Advanced Wellbeing Research Centre, Biomolecular Sciences Research Centre, Sheffield Hallam University, UK; Kara Darling—Lighthouse Complex Care Clinics, USA; Helen Davies—Department of Respiratory Medicine, University Hospital of Wales, Cardiff, Wales, UK; Kate Davies—Long Covid Kids, UK; David Davies-Payne—Starship Children's Hospital, Auckland, New Zealand; Kevin Deans—NHS Grampian & University of Aberdeen, Foresterhill, Aberdeen, UK; Luis Del Carpio Orantes—Study Group for the Diagnosis and Treatment of COVID-19, Veracruz, Mexico; Anne Doherty—Four Peaks Healthcare Associates and the International Association of Chronic fatigue Syndrome/Myalgic Encephalomyelitis, Ireland; Theresa Dowell—Bateman Horne Center, University of Utah, USA; Rae Duncan*—The Newcastle Hospitals NHS Foundation Trust, Newcastle Upon Tyne, UK; The Long COVID Expert Advisory Group, World Health Network; Raechel A. Evans—National Institute for Health Research Biomedical Research Centre, Dept of Respiratory Sciences, University of Leicester, Leicester UK; Andrew Ewing*—World Health Network Long Covid Expert Advisory Group, Department of Chemistry and Molecular Biology, University of Gothenburg, Sweden; Mark Faghy*—Biomedical and Clinical Exercise Science Research Theme, University of Derby, Derby, UK; Emilia Liana Falcone—Center for Immunity, Inflammation and Infectious Diseases, Montreal Clinical Research Institute (IRCM); Department of Microbiology, Infectious Diseases and Immunology, Université de Montréal; Department of Medicine, Université de Montréal; Division of Microbiology and Infectious Diseases, Department of Medicine, Centre Hospitalier de l’Université de Montréal (CHUM), Montreal, QC, Canada; Bingwen Eugene Fan—Department of Haematology, Tan Tock Seng Hospital, 308433; Department of Laboratory Medicine, Khoo Teck Puat Hospital; Lee Kong Chian School of Medicine, Nanyang Technological University; Yong Loo Lin School of Medicine, National University of Singapore, Singapore; Emily Fatakhov—Icahn School of Medicine at Mount Sinai Hospital, NY, USA; Kelly Fearnley—Long COVID Doctors for Action, UK; Artur Fedorowski—Department of Cardiology, Karolinska University Hospital, and Department of Medicine, Karolinska Institute, Stockholm, Sweden; Carlo Ferrarese—Department of Medicine and Surgery and Milan Center for Neuroscience, University of Milano-Bicocca; Department of Neurology, Fondazione IRCCS San Gerardo dei Tintori, Monza, Italy; Francesco Ferraro—Clinical Exercise and Rehabilitation Research Centre, University of Derby, Derby, UK; Gemma Figtree—Kolling Institute, Royal North Shore Hospital and University of Sydney, Sydney, Australia; Laurie Findlay*—Royal North Shore Hospital, Department of Medicine, Sydney, Australia; Alberto Fortini—Internal Medicine, San Giovanni di Dio Hospital, Firenze, Italy; Matteo Foschi—Department of Biotechnological and Applied Clinical Sciences, University of L’Aquila, L’Aquila, Italy; Francois Gagnon—The Other Pain Clinic, Calgary, AB T2P 1K1, Canada; Nick Gall—Consultant Cardiologist (Arrhythmias and Neuro-cardiology), Honorary Senior Lecturer, KCL, Patron, POTS UK, Medical adviser STARS, HMSA, Medical detection dogs; Leo Galland*—Foundation for Integrated Medicine, New York, NY, USA; World Health Network Long Covid Expert Advisory Group; Natasha Gerbis—University of Sydney; Northern Beaches Neurology, NSW, Australia; Ali Gholamrezanezhad—University of Southern California, Los Angeles, CA, USA; Amine Ghram—Department of Cardiac Rehabilitation, Heart Hospital, Hamad Medical Corporation, Doha, Qatar; Research Laboratory "Heart Failure, LR12SP09", Hospital Farhat HACHED of Sousse, Sousse, Tunisia; Healthy Living for Pandemic Event Protection (Hl-Pivot) Network, Chicago, IL, United States; Andréa Lúcia Goncalves da Silva—Universidade de Santa Cruz do Sul – UNISC, Brazil; Helen Goss—Long Covid Kids, Director for Scotland; Stephanie Grach—Mayo Clinic, Division of General Internal Medicine, Rochester, MN, USA; Caroline Gregoire—None listed; Gary Groot—University of Saskatchewan, College of Medicine, Department of Community Health and Epidemiology, Canada; Riccardo Guanà—Riccardo Guanà, MD, PhD, Regina Margherita Children's Hospital, Turin, Italy; Eric Guedj—Service de Médecine Nucléaire, Hôpital de la Timone, CERIMED, Institut Fresnel, Aix Marseille Univ, APHM, CNRS, Centrale Marseille, Marseille, France; Claire Hastie*—Founding Member of the charity Long Covid Support; Merel Hellemons—Erasmus University Medical Center, Pulmonary Medicine, the Netherlands; Melanie Hoppers—The Bateman Horne Center of Excellence, Salt Lake City, UT; Mohammad-Salar Hosseini—Research Center for Integrative Medicine in Aging, Aging Research Institute, Tabriz University of Medical Sciences, Tabriz, Iran; Lee Ingle—School of Sport, Exercise & Rehabilitation Science, University of Hull, UK; Linn Jarte—Swansea Bay University, Anaesthetics Department, Health Board (SBUHB), Wales, UK; David Joffe*—World Health Network Long COVID Expert Advisory Group, Respiratory and Sleep Medicine, Royal North Shore Hospital; Woolcock Institute of Medical Research (Sleep Group), Sydney, Australia; Binita Kane*—Manchester University Foundation Trust, School fo Biological Sciences University of Manchester, UK; Leslie Kasza*—University of Alberta, Canada; David Kaufman—Center for Complex Diseases, Seattle, WA, USA; Sundeep Kaul—Consultant Physician in Respiratory & Intensive Care Medicine, Harefield Hospital, UK; Douglas Kell—Department of Biochemistry, Cell and Systems Biology, Institute of Systems, Molecular and Integrative Biology, University of Liverpool, Crown St, Liverpool, UK; and The Novo Nordisk Foundation Centre for Biosustainability, Technical University of Denmark; Daphne Kemp—Lived Experience of Long Covid; Robin Kerr—NHS Borders, Melrose, Scotland, UK; Asad Khan*—Directorate of Respiratory Medicine, Manchester University Hospitals, North West Lung Centre, Manchester M23 9LT, UK; Abbas Khushnood*—Freeman Hospital, Newcastle,, UK and Long Covid Kids; SangYun Kim—Department of Neurology, Seoul National University College of Medicine; Clinical Neuroscience Center, Seoul National University Bundang Hospital, South Korea; Cheryl Koh—Hollywood GP, Nedlands, WA, Australia; Nik Kudiersky—Sheffield Hallam University, Advanced Wellbeing Research Centre, Sheffield, UK; Katharina Kurz—Department of Internal Medicine II, Innsbruck Medical University, Innsbruck, Austria; Lanserhof Health Resort, Lans, Austria; Gonzalo Labarca—Department of Respiratory Diseases, School of Medicine, Pontificia Universidad Católica de Chile, Santiago, Chile; Amos Lal—Division of Pulmonary, Critical Care and Sleep Medicine, Mayo Clinic, Rochester, MN, USA; Francisco Landi—Fondazione Policlinico Universitario "Agostino Gemelli" IRCCS, Rome, Italy; Dennis Lau—The University of Adelaide and Royal Adelaide Hospital, Adelaide, South Australia, Australia; Jaco Laubscher—Mediclinic Stellenbosch, South Africa; Joshua Leisk—Born Free, NSW, Australia; Jacob Leone—Door One Concierge Naturopathic Medical Group, Novato, CA, USA; Graham Lloyd-Jones—Salisbury NHS Foundation Trust, UK; C. Raina MacIntyre*—Biosecurity Program, Kirby Institute, University of New South Wales, Sydney, Australia; Kushal Madan—Associate Professor, GRIPMER; Director, Cardiac Rehabilitation, Dharma Vira Heart Center, Sir Ganga Ram Hospital, New Delhi, India; Stuart Malcolm—Real Time Health Monitoring, San Francisco, CA, USA; Harsha Master—Hertfordshire Community NHS Trust, UK; Sammie McFarland*—CEO & Founder, Long Covid Kids & Friends, U.K; Gez Medinger—Lived Experience of Long Covid; Yolanda Meije—Infectious Disease Unit—Internal Medicine Department, Hospital de Barcelona, Societat Cooperativa d'Instal·lacions Assistencials Sanitàries (SCIAS), Barcelona, Spain; Alexios-Fotios Mentis—BGI-Shenzhen, Shenzhen, Guangdong, China; Francisco Mera-Cordero—Director Long COVID Blue Healthcare, Madrid, Spain; David Miller—University Hospitals, Connor Whole Health and Rainbow Babies and Children's Hospital, Cleveland OH; Daniel Munblit*—University Hospitals, Connor Whole Health and Rainbow Babies and Children's Hospital, Cleveland OH; Bethan Myers—Haematology Department, University Hospitals of Leicester; Leicester Royal Infirmary, UK; Hiten Naik—Post-COVID-19 Interdisciplinary Clinical Care Network; Department of Medicine, The University of British Columbia, Vancouver, Canada; Javaid Nauman—Department of Circulation and Medical Imaging, Faculty of Medicine and Health Sciences, Norwegian University of Science and Technology (NTNU), Trondheim, Norway; Institute of Public Health, College of Medicine and Health Sciences, United Arab Emirates University, Al-Ain, United Arab Emirates; Jeremy Nicholson*—Director, Australian National Phenome Center, Perth WA; Pro-Vice Chancellor for Health Sciences, Murdoch University, Perth, Western Australia; Adjunct Clinical Professor, Faculty of Health and Medical Sciences, University of Western Australia; Emeritus Professor of Biological Chemistry, Imperial College London; Visiting Professor, Nanyang Technological University, Singapore; Regional Adjunct Professor, Khon Kaen University, Thailand; Josef Niebauer—University Institute of Sports Medicine, Prevention and Rehabilitation, Paracelsus Medical University, Salzburg, Austria; Estefania Nova-Lamperti—Molecular and Translational Immunology Laboratory, Clinical Biochemistry and Immunology Department, Pharmacy Faculty, Universidad de Concepción, Concepción, Chile; Maria Olivier—Dr. Maria Olivier, Inc., Kuils River, South Africa; Otieno Martin Ong'wen—Afyafrica Orthopedic Services; Nairobi, Kenya; Rebecca Owen—University of Derby, College of Science and Engineering, Derby, UK; Kelly O’Brien—Department of Physical Therapy, Temerty Faculty of Medicine, University of Toronto; Rehabilitation Sciences Institute, University of Toronto; Institute of Health Policy, Management and Evaluation (IHPME), Dalla Lana School of Public Health, University of Toronto, Toronto, Ontario, Canada; Stefano Pallanti—Institute for Neurosciences, Florence (Italy), Albert Einstein College of Medicine, NY, USA; Roger Paredes—Department of Infectious Diseases & IrsiCaixa, Hospital Germans Trias i Pujol, Badalona,Catalonia, Spain; Tina Peers—Founder/Managing Director of the Menopause Consultancy and MCAS/Long Covid Clinic, London; Alice Perlowski—Blooming Magnolia, Los Angeles, CA, USA; Colin Pidgeon—Northern Ireland Representative of Long COVID Kids; Julianne Pitzele—The O2 Studio, Southlake, TX, USA; Chris Ponting—Expert in Genetics and Cancer; Anna Porter—NHS England North Central London and The Long Covid Clinic, UK; Etheresia Pretorius*—World Health Network; Department of Physiological Sciences, Faculty of Science, Stellenbosch University, Stellenbosch, WC, South Africa; Department of Biochemistry, Cell and Systems Biology, Institute of Systems, Molecular and Integrative Biology, Faculty of Health and Life Sciences, University of Liverpool, UK; Amy Proal*—PolyBio Research Foundation, USA; Milo Puhan—Epidemiology, Biostatistics und Prevention Institute (EBPI) University of Zurich, Switzerland; David Putrino*—Cohen Center for recovery from complex chronic illness, Department of Rehabilitation and Human Performance, Icahn School of Medicine at Mount Sinai, NY, USA; Shaun Peter Qureshi—Centre for Medical Education, School of Medicine, University of Dundee, Ninewells Hospital, Dundee, DD1 9SY, UK; Sunil Raina—Dr. RP Government Medical College, Tanda (HP), India; Nandini Raj—Parent of Child with Long COVID, Pediatric Long COVID Advocate; Ashwin Rajenesh—N.S. Memorial Institute of Medical Sciences, Kerala, India; Clare Rayner—Society of Occupational Medicine (Long Covid Taskforce), UK; Alfonso J. Rodriguez-Morales—Faculty of Health Sciences, Universidad Científica del Sur, Lima, Peru; Grupo de Investigación Biomedicina, Faculty of Medicine, Fundación Universitaria Autónoma de las Américas-Institución Universitaria Visión de las Américas, Pereira, 660003, Colombia; Luca Roncati—Department of Life Sciences, Health and Healthcare Professions, Link Campus University, Rome, Italy; Alberto Rosario*—World Health Network, Long Covid Advisory Group, Infection Prevention Team; Cilla Rosen—Royal Berkshire Long COVID Service; The Grange Surgery, NHS, UK; Ilene Ruhoy—Medical Director, Chiari/EDS Center, Mount Sinai South, NY, USA; Leslie Rydberg—Department of Physical Medicine and Rehabilitation, Northwestern University Feinberg School of Medicine, Shirley Ryan AbilityLab, Evanston, IL, USA; Špela Šalamon*—World Health Network; Carmen Scheibenbogen—Charité—Universitätsmedizin Berlin; Institut für Med. Immunologie, Berlin, Germany; Sarah Schenck—ChristianaCare, Wilmington, DE, USA; Katharine Seagly—Post-COVID Conditions Clinic, Physical Medicine and Rehabilitiation, University of Michigan, Ann Arbor, MI, USA; Jaffer Shah—Weill Cornell Medicine New York, NY, USA; Ben Sinclair—Founder / Managing Director, Dr. Finlay's Private Practice, The Colmore Building, 20 Colmore Circus, Birmingham B46AT; Ioakim Spyridopoulos—Translational and Clinical Research Institute, Newcastle University, UK; Warren Tate—Department of Biochemistry, School of Biomedical Sciences, University of Otago, Dunedin, New Zealand; Claire Taylor*—Jura health, Perth, Scotland; Catherine Tewolde—Real Time Health Monitoring, San Francisco, CA, USA; Alain Thierry—Institut de Recherche en Cancérologie de Montpellier, INSERM U1194, Université; Montpellier Cancer Institute (ICM), Montpellier, France; Callum Thomas—University of Derby, Derby, UK; Tracey Thompson—Lived Experience of Long Covid; Irene Tosetti—Medgate Group, Switzerland; Eleonora Trecca—IRCCS Casa Sollievo Della Sofferenza, Department of Otorhinolaryngology and Maxillofacial Surgery, San Giovanni Rotondo (Foggia), Italy; Jordan Vaughn—The Microvascular Research Foundation for Spike Protein and Long Covid, USA; Chantelle Venter—Stellenbosch University, South Africa; Amber Vermeesch—UNC Greensboro School of Nursing, NC, USA; Joe Vipond—University of Calgary, Canada; Guiulia Vivaldi—Kings College, London, UK; Maxine Waters—Department of Physiological Sciences, Stellenbosch University, South Africa; William Weir—The ME Association, UK; Emma Weisblatt—University of Cambridge, Dept of Psychology and Cambridgeshire and Peterborough NHS Foundation Trust, UK; Julien Wist*—Head of Operations and Professor in Computational Spectroscopy, Murdoch University, Australia; Visiting Professor, Imperial College London, UK; Professor, Universidad del Valle, Cali, Colombia; May Wong—University of Sydney, Australia; Rob Wüst—Department of Human Movement Sciences, Faculty of Behavioural and Movement Sciences, Vrije Universiteit Amsterdam, Amsterdam Movement Sciences, The Netherlands; Azfar Zaman—Freeman Hospital, Vascular Biology and Medicine, Newcastle University, Newcastle Upon Tyne, UK; James Zhang—Shire Family Medicine, NSW, Australia

*Member of the extended evaluation committee.

The following three panellists declined to be listed in Table S1 as authors.

1 David Fedson, Professor of Medicine (retired), University of Virginia, USA

2 Sarah Glynne, Oxshott Medical Practice, Oxshott Surrey, UK

3 Joan Soriano, no affiliation listed

The correct affiliations for Daniel Munblit^29,46^ should read as follows:

^29^Care for Long Term Conditions Division, Florence Nightingale Faculty of Nursing, Midwifery and Palliative Care, King’s College London, London, UK

^46^Department of Paediatrics and Paediatric Infectious Diseases, Institute of Child’s Health, Sechenov First Moscow State Medical University (Sechenov University), Moscow, Russia
