# Supplementary material for: Long COVID clinical evaluation, research and impact on society: a global expert consensus
Source: Ann Clin Microbiol Antimicrob. 2025 Apr 20;24:27. doi: 10.1186/s12941-025-00793-9 (PMC12010688; doi:10.1186/s12941-025-00793-9)
Supplement: Supplementary file 1 — Additional file 1. [file 12941_2025_793_MOESM1_ESM.pdf]

Long COVID clinical evaluation, research & impact on society: A global expert consensus

Supporting information

Table S1. List of expert panellist authors[1] and affiliations.

#	Last Name	First Name	Affiliation
1	Afrin	Lawrence B.	AIM Center for Personalized Medicine, Purchase, New York, USA
2	Alvarez Moreno	Carlos Arturo	Universidad Nacional de Colombia; Clinica Colsanitas, Colombia
3	Alwan	Nisreen	University of Southampton, UK
4	Amaravadi	Sampath Kumar	School of Sport, Exercise and Rehabilitation Sciences, University of Birmingham, Birmingham, UK
5	Athie-Morales	Veronica	ConCiencia ECAI, Mexico Cty, Mexico
6	Atkinson	Philip	Consultant Occupational Physician, University of Morecambe Bay NHS Trust, UK
7	Axtell	Robert	Southern Connecticut State University, Health & Movement Sciences, CT, USA
8	Ayuzo	Cipatli	Tecnologico de Monterrey, NL Mexico
9	Azola	Alba	Johns Hopkins School of Medicine, Department of Physical Medicine and Rehabilitation, Baltimore, USA
10	Bagley	James	Department of Kinesiology, San Francisco State University, San Francisco, CA, USA
11	Bagnato	Sergio	Unit of Neurophysiology and Unit for Severe Acquired Brain Injuries, Giuseppe Giglio Foundation, Cefalù, Italy
12	Banerjee	Amitava	Institute of Health Informatics, University College London, London, UK
13	**Bar-Yam***	Yaneer	World Health Network, New England Complex Systems Institute, Cambridge, MA
14	Barry	John	Good Shepherd Penn Partners University City, Philadelphia, USA
15	Bassi-Dibai	Daniela	Universidade Cuema y Centro Universitário Santa Terezinha CEST, São Luís, MA, Brazil
16	Bheréur	Anne	Universite de Montreal, Montreal, Quebec, Canada
17	**Bilodeau***	Stephane	World Health Network and Department of Bioengineering, McGill University, Montreal, Canada
18	Bix	Gregory	Tulane University School of Medicine, Clinical Neuroscience Research Center, New Orleans, LA, USA
19	Blanco	Jose-Ramon	Hospital Universitario San Pedro – CIBIR, La Rioja, Spain
20	**Blitshteyn***	Svetlana	Department of Neurology, University at Buffalo Jacobs School of Medicine, Buffalo, NY; Dysautonomia Clinic, Williamsville, NY, USA
21	Bonilla	Hector	Stanford Post-Acute COVID-19 Syndrome Clinic, Palo Alto, CA, USA
22	Bowers	Angela	23 Bowers Angela Southlake Dermatology (Platinum Dermatology Partners), Southlake, TX, USA

#	Last Name	First Name	Affiliation
23	**Brooks***	Anna E.S.	Liggins Institute, The University of Auckland, Auckland, New Zealand; School of Biological Sciences, Faculty of Science, The University of Auckland, Auckland, New Zealand; The Maurice Wilkins Centre, Auckland, New Zealand
24	Bruyneel	Marie	Department of Pneumology, CHU Saint-Pierre, Brussels, Belgium and Université Libre de, Bruxelles, Brussels, Belgium; Department of Pneumology, CHU Brugmann, Brussels, Belgium and Université Libre de Bruxelles, Brussels, Belgium
25	Brugler Yonts	Alexandra	Children's National Hospital/ The George Washington University School of Medicine and Health Sciences in Washington, DC, USA
26	**Buonsenso***	Danilo	Department of Woman and Child Health and Public Health, Fondazione Policlinico Universitario A. Gemelli IRCCS, Rome, Italy, Area Pediatrica, Dipartimento di Scienza Della Vita e Sanità Pubblica, Università Cattolica del Sacro Cuore, 00136 Rome, Italy.
27	Bücker	Gunnar	Nephrologische Praxis in Osnabrück, Germany
28	Capaccione	Kathleen	Department of Radiology, Columbia University, New York, NY, USA
29	Carroll	Harriet	Long Covid Scientific Consultancy, UK; University of Aberdeen, UK; Lund University, Sweden
30	Cathebras	Pascal	Department of internal medicine, University Hospital & Jean-Monnet University, Saint-Etienne, France
31	Chaudhry	Ashish	KD Haemophilia and Thrombosis Centre, Royal Free Hospital, Univ. College London, UK
32	Cheda	Bela	Center for Complex Diseases, Mountain View, CA, USA
33	Chia	Karin	North Shore Private Hospital, St. Leonards, NSW, Australia
34	Chowdary	Pratima	Univ. College London; KD Comprehensive Care Haemophlia Centre; Haemophlia, Rheumatology and Haematology Royal Free London NHS Foundation Trust, UK
35	Chung	Tae Hwan	Johns Hopkins Postural Orthostatic Tachycardia Syndrome (POTS) Program, Lutherville, MD, USA
36	Conner	Leslie	Real Time Health Monitoring, San Francisco, CA, USA
37	Conte	Caterina	IRCCS MultiMedica, Milan, Italy; Università San Raffaele, Rome, Italy
38	**Curtin***	Jennifer	Real Time Health Monitoring, San Francisco, CA, USA
39	Dalton	Caroline	Advanced Wellbeing Research Centre, Biomolecular Sciences Research Centre, Sheffield Hallam University, UK
40	Darling	Kara	Lighthouse Complex Care Clinics, USA
41	Davies	Helen E.	Department of Respiratory Medicine, University Hospital of Wales, Cardiff, Wales, UK
42	Davies	Kate	Long Covid Kids, UK
43	Davies-Payne	David	Starship Children's Hospital, Auckland, New Zealand
44	Deans	Kevin	NHS Grampian & University of Aberdeen, Foresterhill, Aberdeen, UK
45	Del Carpio Orantes	Luis	Study Group for the Diagnosis and Treatment of COVID-19, Veracruz, Mexico
46	Doherty	Anne M.	School of Medicine, University College Dublin, Ireland
47	Dowell	Theresa	Bateman Horne Center, University of Utah, USA
48	**Duncan***	Rae	The Newcastle Hospitals NHS Foundation Trust, Newcastle Upon Tyne, UK; The Long COVID Expert Advisory Group, World Health Network

#	Last Name	First Name	Affiliation
49	Evans	Rachael A.	National Institute for Health Research Biomedical Research Centre, Dept of Respiratory Sciences, University of Leicester, Leicester UK
50	**Ewing***	Andrew	World Health Network Long Covid Expert Advisory Group, Department of Chemistry and Molecular Biology, University of Gothenburg, Sweden
51	**Faghy***	Mark	Biomedical and Clinical Exercise Science Research Theme, University of Derby, Derby, UK
52	Falcone	Emilia Liana	Center for Immunity, Inflammation and Infectious Diseases, Montreal Clinical Research Institute (IRCM); Department of Microbiology, Infectious Diseases and Immunology, Université de Montréal; Department of Medicine, Université de Montréal; Division of Microbiology and Infectious Diseases, Department of Medicine, Centre Hospitalier de l'Université de Montréal (CHUM), Montreal, QC, Canada.
53	Fan	Bingwen Eugene	Department of Haematology, Tan Tock Seng Hospital, 308433; Department of Laboratory Medicine, Khoo Teck Puat Hospital; Lee Kong Chian School of Medicine, Nanyang Technological University; Yong Loo Lin School of Medicine, National University of Singapore, Singapore
54	Fatakhov	Emily	Icahn School of Medicine at Mount Sinai Hospital, NY, USA
55	Fearnley	Kelly	Long COVID Doctors for Action, UK
56	Fedorowski	Artur	Department of Cardiology, Karolinska University Hospital, and Department of Medicine, Karolinska Institute, Stockholm, Sweden
57	Ferrarese	Carlo	Department of Medicine and Surgery and Milan Center for Neuroscience, University of Milano-Bicocca; Department of Neurology, Fondazione IRCCS San Gerardo dei Tintori, Monza, Italy
58	Ferraro	Francesco	Clinical Exercise and Rehabilitation Research Centre, University of Derby, Derby, UK
59	Figtree	Gemma	Kolling Institute, Royal North Shore Hospital and University of Sydney, Sydney, Australia
60	Findlay*	Laurie	Royal North Shore Hospital, Department of Medicine, Sydney, Australia
61	Fortini	Alberto	Internal Medicine, San Giovanni di Dio Hospital, Firenze, Italy
62	Foschi	Matteo	Department of Biotechnological and Applied Clinical Sciences, University of L'Aquila, L'Aquila, Italy
63	Gagnon	Francois	The Other Pain Clinic, Calgary, AB T2P 1K1, Canada
64	Gall	Nick	Consultant Cardiologist (Arrhythmias and Neuro-cardiology), Honorary Senior Lecturer, KCL, Patron, POTS UK, Medical adviser STARS, HMSA, Medical detection dogs
65	**Galland***	Leo	Foundation for Integrated Medicine, New York, NY, USA; World Health Network Long Covid Expert Advisory Group
66	Gerbis	Natasha	University of Sydney; Northern Beaches Neurology, NSW, Australia
67	Gholamrezanezhad	Ali	University of Southern California, Los Angeles, CA, USA
68	Ghram	Amine	Department of Cardiac Rehabilitation, Heart Hospital, Hamad Medical Corporation, Doha, Qatar; Research Laboratory "Heart Failure, LR12SP09", Hospital Farhat HACHED of Sousse, Sousse, Tunisia; Healthy Living for Pandemic Event Protection (Hl-Pivot) Network, Chicago, IL, United States
69	Goncalves da Silva	Andréa Lúcia	Universidade de Santa Cruz do Sul – UNISC, Brazil

#	Last Name	First Name	Affiliation
70	Goss	Helen	Helen Goss, Long Covid Kids, Director for Scotland
71	Grach	Stephanie	Mayo Clinic, Division of General Internal Medicine, Rochester, MN, USA
72	Gregoire	Caroline	MD with lived experience with Long Covid
73	Groot	Gary	University of Saskatchewan, College of Medicine, Department of Community Health and Epidemiology, Canada
74	Guanà	Riccardo	Riccardo Guanà, MD, PhD, Regina Margherita Children's Hospital, Turin, Italy
75	Guedj	Eric	Service de Médecine Nucléaire, Hôpital de la Timone, CERIMED, Institut Fresnel, Aix Marseille Univ, APHM, CNRS, Centrale Marseille, Marseille, France
76	Harahsheh	Ashraf	Division of Cardiology, Children's National Heart Center, Children's National Hospital, Department of Pediatrics, The George Washington University School of Medicine & Health Sciences, Washington, DC, USA
77	**Hastie***	Claire	Founding Member of the charity Long Covid Support
78	Hellemons	Merel	Erasmus University Medical Center, Pulmonary Medicine, the Netherlands
79	Hoppers	Melanie	The Bateman Horne Center of Excellence, Salt Lake City, UT
80	Hosseini	Mohammad-Salar	Research Center for Integrative Medicine in Aging, Aging Research Institute, Tabriz University of Medical Sciences, Tabriz, Iran
81	Ingle	Lee	School of Sport, Exercise & Rehabilitation Science, University of Hull, UK
82	Järte	Linn	Anaesthetics department, Swansea Bay University Health Board, Wales, UK
83	**Joffe***	David	World Health Network Long COVID Expert Advisory Group, Respiratory and Sleep Medicine, Royal North Shore Hospital; Woolcock Institute of Medical Research (Sleep Group), Sydney, Australia
84	**Kane***	Binita	Manchester University Foundation Trust, School for Biological Sciences University of Manchester, UK
85	**Kasza***	Leslie	University of Alberta, Canada
86	Kaufman	David	Center for Complex Diseases, Seattle, WA, USA
87	Kaul	Sundeep	Consultant Physician in Respiratory & Intensive Care Medicine, Harefield Hospital, UK
88	Kell	Douglas	Department of Biochemistry, Cell and Systems Biology, Institute of Systems, Molecular and Integrative Biology, University of Liverpool, Crown St, Liverpool, UK; and The Novo Nordisk Foundation Centre for Biosustainability, Technical University of Denmark
89	Kemp	Daphne	Lived Experience of Long Covid
90	Kerr	Robin	NHS Borders, Melrose, Scotland, UK
91	**Khan***	Asad	Directorate of Respiratory Medicine, Manchester University Hospitals, North West Lung Centre, Manchester M23 9LT, UK
92	**Khushnood***	Abbas	Freeman Hospital, Newcastle,, UK and Long Covid Kids
93	Kim	SangYun	Department of Neurology, Seoul National University College of Medicine; Clinical Neuroscience Center, Seoul National University Bundang Hospital, South Korea
94	Koh	Cheryl	Hollywood GP, Nedlands, WA, Australia
95	Kudiersky	Nik	Sheffield Hallam University, Advanced Wellbeing Research Centre, Sheffield, UK
96	Kurz	Katharina	Department of Internal Medicine II, Innsbruck Medical University, Innsbruck, Austria; Lanserhof Health Resort, Lans, Austria

#	Last Name	First Name	Affiliation
97	Labarca	Gonzalo	Department of Respiratory Diseases, School of Medicine, Pontificia Universidad Católica de Chile, Santiago, Chile
98	Lal	Amos	Division of Pulmonary, Critical Care and Sleep Medicine, Mayo Clinic, Rochester, MN, USA
99	Landi	Francesco	Fondazione Policlinico Universitario "Agostino Gemelli" IRCCS, Rome, Italy
100	Lau	Dennis	The University of Adelaide and Royal Adelaide Hospital, Adelaide, South Australia, Australia
101	Laubscher	Jaco	Mediclinic Stellenbosch, South Africa
102	Leisk	Joshua	Born Free, NSW, Australia
103	Leone	Jacob	Door One Concierge Naturopathic Medical Group, Novato, CA, USA
104	Lloyd-Jones	Graham	Salisbury NHS Foundation Trust, UK
105	**MacIntyre***	C. Raina	Biosecurity Program, Kirby Institute, University of New South Wales, Sydney, Australia
106	Madan	Kushal	Professor, GRIPMER; Director, Cardiac Rehabilitation, Dharma Vira Heart Center, Sir Ganga Ram Hospital, New Delhi, India
107	Malcolm	Stuart	Real Time Health Monitoring, San Francisco, CA, USA
108	Master	Harsha	Hertfordshire Community NHS Trust, UK
109	**McFarland***	Sammie	CEO & Founder, Long Covid Kids & Friends, U.K.
110	Medinger	Gez	Lived Experience of Long Covid
111	Meije	Yolanda	Infectious Disease Unit - Internal Medicine Department, Hospital de Barcelona, Societat Cooperativa d'Instal·lacions Assistencials Sanitàries (SCIAS), Barcelona, Spain
112	Mentis	Alexios-Fotios A.	BGI-Shenzhen, Shenzhen, 518083 Guangdong, China
113	Mera-Cordero	Francisco	Director Long COVID Blue Healthcare, Madrid, Spain
114	Miller	David	University Hospitals, Connor Whole Health and Rainbow Babies and Children's Hospital, Cleveland OH
115	**Munblit***	Daniel	Imperial College and King's College, UK
116	Myers	Bethan	Haematology Department, University Hospitals of Leicester; Leicester Royal Infirmary, UK
117	Naik	Hiten	Post-COVID-19 Interdisciplinary Clinical Care Network; Department of Medicine, The University of British Columbia, Vancouver, Canada
118	Nauman	Javaid	Department of Circulation and Medical Imaging, Faculty of Medicine and Health Sciences, Norwegian University of Science and Technology (NTNU), Trondheim, Norway; Institute of Public Health, College of Medicine and Health Sciences, United Arab Emirates University, Al-Ain, United Arab Emirates.
119	**Nicholson***	Jeremy	Director, Australian National Phenome Center, Perth WA; Pro-Vice Chancellor for Health Sciences, Murdoch University, Perth, Western Australia; Adjunct Clinical Professor, Faculty of Health and Medical Sciences, University of Western Australia; Emeritus Professor of Biological Chemistry, Imperial College London; Visiting Professor, Nanyang Technological University, Singapore; Regional Adjunct Professor, Khon Kaen University, Thailand
120	Niebauer	Josef	University Institute of Sports Medicine, Prevention and Rehabilitation, Paracelsus Medical University, Salzburg, Austria

#	Last Name	First Name	Affiliation
121	Nova-Lamperti	Estefania	Molecular and Translational Immunology Laboratory (ATE220034), Immunology Department, Pharmacy Faculty, Universidad de Concepción, Concepción, Chile
122	Olivier	Maria	Dr. Maria Olivier, Inc., Kuils River, South Africa
123	Ong'wen	Otieno Martin	Afyafrica Orthopedic Services; Nairobi, Kenya
124	Owen	Rebecca	University of Derby, College of Science and Engineering, Derby, UK
125	O'Brien	Kelly	Department of Physical Therapy, Temerty Faculty of Medicine, University of Toronto; Rehabilitation Sciences Institute, University of Toronto; Institute of Health Policy, Management and Evaluation (IHPME), Dalla Lana School of Public Health, University of Toronto, Toronto, Ontario, Canada
126	Pallanti	Stefano	Institute for Neurosciences, Florence (Italy), Albert Einstein College of Medicine, NY, USA
127	Paredes	Roger	Department of Infectious Diseases & IrsiCaixa, Hospital Germans Trias i Pujol, Badalona,Catalonia, Spain
128	Peers	Tina	Founder/Managing Director of the Menopause Consultancy and MCAS/Long Covid Clinic, London
129	Perlowski	Alice	Blooming Magnolia, Los Angeles, CA, USA
130	Pidgeon	Colin	Northern Ireland Representative of Long COVID Kids
131	Pitzele	Julianne	The O2 Studio, Southlake, TX, USA
132	Ponting	Chris	Expert in Genetics and Cancer
133	Porter	Anna	NHS England North Central London and The Long Covid Clinic, UK
134	**Pretorius***	Etheresia	World Health Network; Department of Physiological Sciences, Faculty of Science, Stellenbosch University, Stellenbosch, WC, South Africa; Department of Biochemistry, Cell and Systems Biology, Institute of Systems, Molecular and Integrative Biology, Faculty of Health and Life Sciences, University of Liverpool, UK
135	**Proal***	Amy	PolyBio Research Foundation, USA
136	Puhan	Milo	Epidemiology, Biostatistics und Prevention Institute (EBPI) University of Zurich, Switzerland
137	**Putrino***	David	Cohen Center for recovery from complex chronic illness, Department of Rehabilitation and Human Performance, Icahn School of Medicine at Mount Sinai, NY, USA
138	Qureshi	Shaun Peter	Centre for Medical Education, School of Medicine, University of Dundee, Ninewells Hospital, Dundee, DD1 9SY, UK
139	Raina	Sunil	Dr. RP Government Medical College, Tanda (HP), India
140	Raj	Nandini	Parent of Child with Long COVID, Pediatric Long COVID Advocate
141	Rajenesh	Ashwin	N.S. Memorial Institute of Medical Sciences, Kerala, India
142	Rayner	Clare	Society of Occupational Medicine (Long Covid Taskforce), UK
143	Rodriguez-Morales	Alfonso J.	Faculty of Health Sciences, Universidad Científica del Sur, Lima, Peru; Grupo de Investigación Biomedicina, Faculty of Medicine, Fundación Universitaria Autónoma de las Américas-Institución Universitaria Visión de las Américas, Pereira, 660003, Colombia
144	Roncati	Luca	Department of Life Sciences, Health, and Health Care Professions, Link Campus University, Rome, Italy
145	**Rosario***	Alberto	World Health Network, Long Covid Advisory Group, Infection Prevention Team

#	Last Name	First Name	Affiliation
146	Rosen	Cilla	Royal Berkshire Long COVID Service; The Grange Surgery, NHS, UK
147	Ruhoy	Ilene	Medical Director, Chiari/EDS Center, Mount Sinai South, NY, USA
148	Rydberg	Leslie	Department of Physical Medicine and Rehabilitation, Northwestern University Feinberg School of Medicine, Shirley Ryan AbilityLab, Evanston, IL, USA
149	**Šalamon***	Špela	World Health Network
150	Scheibenbogen	Carmen	Charité - Universitätsmedizin Berlin; Institut für Med. Immunologie, Berlin, Germany
151	Schenck	Sarah	ChristianaCare, Wilmington, DE, USA
152	Seagly	Katharine	Post-COVID Conditions Clinic, Physical Medicine and Rehabilitiation, University of Michigan, Ann Arbor, MI, USA
153	Shah	Jaffer	Weill Cornell Medicine New York, NY, USA
154	Sinclair	Ben	Founder Long Covid Clinic, Dr. Finlay's Private Practice, 36 Great Titchfield street, London W1W 8BQ
155	Spyridopoulos	Ioakim	Translational and Clinical Research Institute, Newcastle University, UK
156	Tate	Warren	Department of Pathology, Dunedin School of Medicine, University of Otago, Dunedin, New Zealand
157	**Taylor***	Claire	Jura health, Perth, Scotland
158	Tewolde	Catherine	Real Time Health Monitoring, San Francisco, CA, USA
159	Thierry	Alain	Institut de Recherche en Cancérologie de Montpellier, INSERM U1194, Université; Montpellier Cancer Institute (ICM), Montpellier, France
160	Thomas	Callum	University of Derby, Derby, UK
161	Thompson	Tracey	Lived Experience of Long Covid
162	Tosetti	Irene	Medgate Group, Switzerland
163	Trecca	Eleonora	IRCCS Casa Sollievo Della Sofferenza, Department of Otorhinolaryngology and Maxillofacial Surgery, San Giovanni Rotondo (Foggia), Italy
164	Vaughn	Jordan	The Microvascular Research Foundation for Spike Protein and Long Covid, USA
165	Venter	Chantelle	Stellenbosch University, South Africa
166	Vermeesch	Amber	UNC Greensboro School of Nursing, NC, USA
167	Vipond	Joe	University of Calgary, Canada
168	Vivaldi	Guiulia	Kings College, London, UK
169	Waters	Maxine	Department of Physiological Sciences, Stellenbosch University, South Africa
170	Weir	William	The ME Association, UK
171	Weisblatt	Emma	University of Cambridge, Dept of Psychology and Cambridgeshire and Peterborough NHS Foundation Trust, UK
172	**Wist***	Julien	Head of Operations and Professor in Computational Spectroscopy, Murdoch University, Australia; Visiting Professor, Imperial College London, UK; Professor, Universidad del Valle, Cali, Colombia
173	Wong	May	University of Sydney, Australia
174	Wüst	Rob C.I.	Department of Human Movement Sciences, Faculty of Behavioural and Movement Sciences, Vrije Universiteit Amsterdam, Amsterdam Movement Sciences, The Netherlands
175	Zaman	Azfar	Freeman Hospital, Vascular Biology and Medicine, Newcastle University, Newcastle Upon Tyne, UK

#	Last Name	First Name	Affiliation
176	Zhang	James	Shire Family Medicine, NSW, Australia

*Member of the extended evaluation committee.

[1]The following three panellists declined to be listed in Table S1 as authors.

1 David Fedson, Professor of Medicine (retired), University of Virginia, USA
2 Sarah Glynne, Oxshott Medical Practice, Oxshott Surrey, UK
3 Joan Soriano, no affiliation listed

Table S2. List of Delphi-style statements that did not reach consensus

Round 2

Defining Long COVID as the persistence of symptoms beyond 8 weeks from the acute infection, with no other explanation is also a valid definition of Long COVID.

The definition of Long COVID should also include exacerbation of preexisting conditions following COVID-19 (e.g. flares of pre-existing autoimmune disease, worsening of postural orthostatic tachycardia syndrome [POTS], viral reactivation, worsening of asthma and allergies).

Long COVID should be defined differently for children and adults.

The definition of Long COVID in children should recognise an impairment in day-to-day functioning (different from the Delphi definition for adults).

Long COVID biomarkers are important for diagnosis of the disease, despite its complexity.

In clinical assessment of Long COVID, it is important to perform cardiopulmonary exercise testing to evaluate respiratory capacity.

In treating Long COVID it is important to consider treatment of SARSCoV-2 viral persistence with antiviral medication.

Antidepressant medications can be useful for treatment of Long COVID.

In treating Long COVID it is important to consider use of anti-platelet drugs.

Statins can be useful to treat Long COVID.

Smell training, vitamin A (oil nasal drops), and steroid nasal rinses can be useful for treating olfactory dysfunction in Long COVID.

Pharmacological agents like bupropion, venlafaxine, modafinil or amantadine can be useful for treating Long COVID symptoms.

Selective serotonin re-uptake inhibitors (SSRIs) can be useful for treating Long COVID patients.

Yoga and Tai Chi can be useful in treating some Long COVID patients.

The use of combined nattokinase and/or serrapeptase can be useful in treating some Long COVID patients.

Hyperbaric oxygen therapy can be useful in treating some Long COVID patients.<br><br>Paxlovid can be useful in treating some Long COVID patients.<br><br>Duloxetine can be effective in treating peripheral neuropathy Long COVID.<br><br>Re-imaging can be useful to measure the effect of Long COVID treatment.<br><br>Repeating the cardiopulmonary exercise test (CPET) can be useful to measure the effect of Long COVID treatment.<br><br>Long COVID can improve with vaccination.<br><br>**Round 3**<br><br>Because of the heterogeneity of health problems that occur after COVID-19, the term “Post COVID Conditions” should be used instead of “Long COVID,” even if SARS-CoV2 viral/antigen persistence is a potential cause of some of those health problems.<br><br>Long COVID includes the exacerbation of pre-existing symptoms or a pre-existing medical condition for at least 8-12 weeks after COVID-19.<br><br>Long COVID includes abnormalities in laboratory tests (i.e. liver or kidney function tests) that appear during or within 3 months of acute COVID-19 and persist for at least 8-12 weeks, whether or not there are symptoms or functional disabilities.<br><br>Long COVID includes new onset of a specific disease entity i.e. with a pre-pandemic ICD code, that starts within 3 months after acute COVID-19. Examples include diabetes, hypertension, migraine headache, and autoimmune or allergic diseases.<br><br>The criteria for diagnosing Long COVID in adults should be the same as the criteria for diagnosing Long COVID in children.<br><br>The diagnosis of Long COVID requires that the person have laboratory confirmation of COVID-19 infection during a period of 4-8 weeks prior to the onset of symptoms.<br><br>Evaluation of gut / nasal / oral / vaginal microbiome dysbiosis, with a particular focus on acetaldehyde producing vs. metabolising species, should be investigated as a diagnostic tool for Long COVID.<br><br>Pharmacological agents that promote wakefulness and/or are CNS stimulants can be useful for managing fatigue, hypersomnolence, and cognitive dysfunction in Long COVID patients.<br><br>Yoga and Tai Chi can be useful in managing symptoms in some patients with Long COVID.<br><br>Electronic devices that stimulate the Vagus nerve can be useful in treating patients with Long COVID.<br><br>Melatonin can be useful as an anti-inflammatory and antioxidative agent in patients with Long COVID.<br><br>Hyperbaric oxygen therapy can be useful in treating a subgroup of Long COVID patients with cognitive dysfunction and evidence of end-organ hypoxia e.g. decreased left ventricular function.<br><br>Certain professions (e.g. airline pilots, medical professionals, professional drivers) should be routinely tested for Long COVID-induced cognitive impairment after infection and reinfection if validated tests become clinically available.

Table S3. Summary of Approaches to Treatment and Evaluation Derived from the Consensus and Listed by Topic and Consensus Level

Expert Views on Treatment Recommendations	Consensus Level
Tailored to history and physical examination	A
Multidisciplinary team management	A
Treatment for POTS	A
Sleep disturbance/disorders	A
Identification of new onset diabetes dyslipidaemia, and modification of previous pre-existing comorbidities	A
New onset hypertension	A
Psychological support and evaluation of mental health with targeted treatments of the pathophysiology	A
MOCA and MMSE are NOT appropriate. More comprehensive and potentially repeated neurocognitive evaluation are required	A
Fatigue assessment tools (e.g. Epworth Sleep Scale, Fatigue Severity Scale, etc)	A
Paediatric patients outcomes including school attendance, resuming sport, musical and other activities at a previous level, should considered as good measure of treatment success	A
Treatment of clotting abnormalities	B
Treatment of mast cell activation	B
Treatment of resting tachycardia	B
Treatment of myocarditis and /or pericarditis	B
Treatment of gut dysbiosis	B

Specific Drug Recommendations	
Modulation of autonomic nervous system (e.g. Midodrine, Ivabradine, Beta-blockers)	B
Non-opioid drugs for small fibre neuropathic pain	B
Antidepressants in conjunction with other psychological therapies	B
Therapies targeted at endothelial injury	B
Anticoagulants in a *sub-population* where appropriate diagnostic tests for thrombotic endotheliitis are available *when overseen by an experienced clinician*	B
Adjunctive Therapeutic Recommendations	
Wearable devices to track heart rate variability	B
Cardiac and Respiratory pathology should be excluded prior to recommending graded exercise testing	B
Additional therapies that might be considered	
Biomarkers are important for treatment	C
Pain medication can be useful	C
Nutritional and dietary changes (e.g. Probiotics Vit B Complexes	C
Graded exercise in those with POTS and or PESE	C
Pulmonary rehabilitation	C
Melatonin for Long COVID insomnia	C

**Vagus nerve therapies (e.g. cold exposure, breathwork, mindfulness, compression wear, trauma release, vagus nerve stimulators)**	C
**Anticoagulant-antiplatelet drugs**	C
**Medications for gastroparesis and hyperacidity**	C
**Treatment of viral persistence with anti-viral drugs**	C
**Repeated haemostatic tests for coagulopathy can be useful to measure the effect of Long COVID treatment**	C
**Evaluation Tools**	
**MOCA and MMSE are NOT appropriate. More comprehensive and potentially repeated neurocognitive evaluation are required**	A
**Fatigue assessment tools (e.g. Epworth Sleep Scale, Fatigue Severity Scale, etc)**	A
**Paediatric patients outcomes including school attendance, resuming sport, musical and other activities at a previous level, should considered as good measure of treatment success**	A
**Symptom Burden Questionnaire for Long COVID (SBQ-LC) can be used to monitor the effect of treatment in patients with Long COVID**	B
**Re-imaging for specific Long COVID complications such as pulmonary embolism, myocarditis, and heart failure can be useful to monitor the response to treatment in patients with Long COVID**	B
**If available, repeat measurement of markers of endothelial dysfunction, platelet hyperactivation and abnormal clotting physiology (such as vWF, sCD40 ligand, VEGF & micro-clot detection) can be useful to track the effect of some Long COVID treatments**	B

**If determined safe and appropriate following detailed screening for post-exertional malaise (PEM), repeat cardiopulmonary exercise testing (CPET) can be important to monitor changes in VO2 max and anaerobic threshold and to measure the effectiveness of treatments (including rehabilitation programmes) in Long COVID**	B

Table S4. List of preliminary round questions

A Consensus on best practices in diagnosis and treatment of Long COVID

QUESTIONS (initial round to build consensus statements)
PLEASE NOTE: Sections A-C are for physicians treating Long COVID patients. Researchers, please answer applicable questions in A and D.

A) General Questions

A1) How many patients with Long COVID/post-COVID complications do you treat monthly?
New patients ___
Recurring patients ___

A2) Which definition of Long COVID do you use for diagnosis?
WHO definition ___
Time from infection ___
Other __________

A3) What terminology do you use describe the illness?
PASC ___
PCS ___
Long-COVID ___
Other ________

A4) Please indicate if you are practicing as a (mark all that apply)
private physician ___, a public ___ physician, or a researcher ___

A5) Gender, ethnicity, age, continent? Optional.
F __ M __
Ethnicity: _________
Age: ___
Continent of residence: ______

A6) Do you ask patients if they have had acute COVID, if they have been vaccinated, and when? Yes __ No __

A7) Do you record current medications both prescribed and over the counter? Yes __ No __

A8) Do you record underlying or previously diagnosed medical conditions when diagnosing long COVID? Yes __ No __

A9) After filling out this questionnaire, please tell us what other topics should we include in these questions?

B) Questions about diagnosis of Long COVID

B1) Do you use any long-COVID-specific biomarkers to help confirm diagnosis? Yes __ No __
Please check those you use.
Platelet activation markers ___
Microclotting ___
Cytokines ___
Other ___, please list ______________________

B2) Do you use validated questionnaires? Yes __ No __
If so, please list ________________

B3) Do you use a functional ability scale?
Yes __ No __
If so, please list ________________

B4) Do you perform neurocognitive function tests? Yes __ No __
If yes, please list ____________________________

B5) Do you perform a bedside test to diagnose orthostatic intolerance?
Yes __ No __

B6) Do you screen for organ, circulatory, or immune dysfunction?
Yes __ No __

B7) The following is a list of symptoms by area of the body. Please indicate with an x those which you routinely screen for or ask new patients about.

Body System
General
Post-exertional malaise ___
Fatigue and functional ability and post-exertional symptom exacerbation (PESE) ___
Persistent fevers ___

Neurological/Brain
Sleep disruption ___
Headache ___
Seizures ___
Cognitive dysfunction ("brain fog') ___)___
Mood changes ___
Depersonalisation ___
Vision/eyes ___
Light sensitivity ___
Sound sensitivity ___
Hearing ___
Vertigo ___
Loss of / altered sense of smell ___
Voice / phonation ___
Dry eyes / dry mouth ___
Facial weakness or loss of sensation in face? ___?___
Swallowing ___
Temperature dysregulation ___
Changes in sweating ___

Cardiorespiratory
Dizziness on standing ___
Palpitations ___

Blood pooling in limbs ___
Chest pain ___
SOB ___
Pins and needles in hands/feet or around lips ___

Vascular
Limb pain or heaviness ___
Nail changes ___
Raynaud's ___
Hair loss ___
'Blotchy skin' ___
Vascular rash ___
COVID toes ___

Gastrointestinal
Changes in bowel habits ___
Abdominal pain ___
Food regurgitation / vomiting ___
New food intolerances ___
Changes in weight ___

Allergy
Skin rashes ___
Joint pain ___
Muscle aches pain ___
Respiratory ___

Genitourinary
Sexual function ___
Changes in menstruation
(if applicable) ___)___
Loss of libido ___
Erectile dysfunction ___

Urinary
Bladder dysfunction ___

Endocrine
Tremors (sign of thyroiditis) ___
Polyuria/polydipsia (diabetes) ___

OTHER

Please describe any other symptoms not captured above:

B8) Do you use imaging studies? Yes __ No __
If yes, please check those used.
MRI ___
Xray ___
CT ___
Ultrasound ___
PET Scanning ___
Elastography ___
Other, please list _____________

C) Questions concerning treatment of Long COVID

C1) Which conditions do you treat for Long COVID
MCAS ___
POTS ___
Abnormal clotting pathology ___
Viral persistence ___
Other _________

C2) Do you use other treatments not included above (for example: graded exercise, antidepressants, etc). Yes __ No __
If so, please list.

C3) Which treatments do you find lead to the best patient outcomes?

C4) Is there a treatment you would like to use, but you are unable to for any reason? Yes __ No __
If so, what is it?

C5) Do you measure the effect of treatment? Yes __ No __
If so, how? (Please give examples).

C6) Do you refer patients for sleep evaluation and treatment? Yes __ No __

D) Questions related to research of Long COVID

D1) Where should long COVID research be focused? Check as many as apply.
Vascular ___
Neurological ___
Immune system ___
Autoimmunity ___
Effect of reinfection ___
Antivirals ___
Long COVID in children ___
Sleep ____
Hypermobility ____
Exercise physiology ___
Other (please list) _________

D2) What should be researched in the immune system related to long COVID? Please check all that apply.
Role of cytokines ___
T cells, B cells ___
Dendritic cells ___
Mast cells ___
Platelets ___
Microclots ___
Endothelial dysfunction ___
Microglia and blood brain barrier ___
Hyperlipidaemia ___
Inflammation ___
Other, please list ______________

D3) What organs or systems appear to be affected in Long COVID, but do not show up on initial symptoms? Please list all that apply.
For example,
Liver ___
Pancreas ___
Kidneys ___
Skin ___
Autonomic nervous system ___

Long-term heart or lung disease ___
Reproductive organs ___
Other, please list _______________

D4) What aspects of long COVID should be researched in specifically in children vs adults? Check as many as apply.
Impact on education ___
PANS ___
Longevity of the problem ___
Effect of vaccine on long COVID prevalence and severity ___
Psychological issues ___
ADHD ___
Diabetes ___
Other, please list _______________

D5) Should the importance of previous, ongoing, or coinfections with other pathogens on long COVID be a major research area? Yes __ No __

D6) What are the most important research issues to address with long COVID.
Developing approaches to stop transmission __
Developing new/better approaches to treat the disease __
Please comment __________________________

D7) Should governments or corporate entities contribute to the funding of long COVID research? Yes __ No __

Table S5. List of Statements and open questions for round 2

I. <u>**Background**</u>

1. Your specific responses will remain confidential. Your name and affiliation will only be used to identify you as a respondent to the executive committee. When the manuscript is written, you be given a chance to comment and will need to consent to being a coauthor.
   Name:
   Affiliation:

2. What are your key areas of expertise (mark more than one if appropriate):
A. Scientific research
B. Lived experience of Long COVID
C. Carer/family member of a person with long COVID
D. Clinical management
E. Allied health services

Answer question 3 only If you are involved in clinical management of Long COVID

3. Tell us how many Long COVID patients per week you diagnose and/or treat.
A. None
B. 1-10
C. 11-25
D. 26-40
E. More than 40

II. Long COVID definition

4. The WHO defines Long COVID as "the continuation or development of new symptoms 3 months after the initial SARS-CoV-2 infection, with these symptoms lasting for at least 2 months with no other explanation."
This is a valid definition of Long COVID.
A. Strongly agree
B. Agree
C. Neither agree nor disagree
D. Disagree
E. Strongly disagree

5. Defining Long COVID as the persistence of symptoms beyond 8 weeks from the acute infection, with no other explanation is also a valid definition of Long COVID.
A. Strongly agree
B. Agree
C. Neither agree nor disagree
D. Disagree
E. Strongly disagree

6. The definition of Long COVID should also include exacerbation of pre-existing conditions following COVID-19 (e.g. flares of pre-existing auto-immune disease, worsening of postural orthostatic tachycardia syndrome [POTS], viral reactivation, worsening of asthma and allergies).
A. Strongly agree
B. Agree
C. Neither agree nor disagree
D. Disagree
E. Strongly disagree

7. The Long COVID definition should also include a criterion for significant functional impairment from baseline (including reduction in effort tolerance, even without additional new symptoms)
A. Strongly agree
B. Agree
C. Neither agree nor disagree
D. Disagree
E. Strongly disagree

At the present time, there are different long COVID definitions for adults and children. The current Delphi research definition for Post COVID-19 Condition in children, as published by Stephenson et al. (2022) is: *Post-COVID-19 condition occurs in young people with a history of confirmed SARS-CoV-2 infection, with at least one persisting physical symptom for a minimum duration of 12 weeks after initial testing that cannot be explained by an alternative diagnosis. The symptoms have an impact on everyday functioning, may continue or develop after COVID infection, and may fluctuate or relapse over time. The positive COVID-19 test referred to in this definition can be a lateral flow antigen test, a PCR test or an antibody test*.
Please indicate your level of agreement with the following statements:

8. Long COVID should be defined differently for children and adults.
A. Strongly agree
B. Agree
C. Neither agree nor disagree
D. Disagree
E. Strongly disagree

9. The definition of Long COVID in children should recognise an impairment in day-to-day functioning (different from the Delphi definition for adults).
A. Strongly agree
B. Agree
C. Neither agree nor disagree
D. Disagree
E. Strongly disagree

10. What do you think the ideal of long-covid definition should be?
Please comment: ___________________________________

11. Do you have any further comments on the background and definition of Long COVID?
Comment: ___________________________________

III. <u>Diagnosis and clinical assessment of Long Covid</u>

12. Long COVID biomarkers are important for <u>diagnosis</u> of the disease, despite its complexity.
A. Strongly agree
B. Agree
C. Neither agree nor disagree
D. Disagree
E. Strongly disagree

13. Validated questionnaires are important in the <u>assessment</u> of Long COVID.
A. Strongly agree
B. Agree

C. Neither agree nor disagree
D. Disagree
E. Strongly disagree

14. A functional ability/capacity evaluation is important in the assessment of Long COVID.
A. Strongly agree
B. Agree
C. Neither agree nor disagree
D. Disagree
E. Strongly disagree

15. Neurocognitive testing is important for the assessment of Long COVID.
A. Strongly agree
B. Agree
C. Neither agree nor disagree
D. Disagree
E. Strongly disagree

16. It is important to take into account a person's self-reported cognitive difficulties even if their performance on standard cognitive tests is within normal limits.
A. Strongly agree
B. Agree
C. Neither agree nor disagree
D. Disagree
E. Strongly disagree

17. Clinical assessment of Long COVID should examine the change in a person's functional abilities from their previous baseline, as well as the cost of maintaining functioning (e.g. more effort and/or time required, and physical or cognitive exhaustion following tasks).
A. Strongly agree
B. Agree
C. Neither agree nor disagree
D. Disagree
E. Strongly disagree

18. Bedside tests to diagnose orthostatic intolerance are important in the assessment of Long COVID.
A. Strongly agree
B. Agree
C. Neither agree nor disagree
D. Disagree
E. Strongly disagree

19. Screening for organ and circulatory dysfunction is important in the assessment and management of Long COVID.
A. Strongly agree
B. Agree
C. Neither agree nor disagree
D. Disagree
E. Strongly disagree

20. Screening for immune dysfunction/dysregulation is important in the assessment and management of Long COVID.
A. Strongly agree
B. Agree
C. Neither agree nor disagree
D. Disagree
E. Strongly disagree

21. In diagnosing Long COVID patients it is important to screen for <u>general</u> symptoms including fatigue, post-exertional malaise (PEM)/post-exertional symptom exacerbation (PESE), and persistent fevers.
A. Strongly agree
B. Agree
C. Neither agree nor disagree
D. Disagree
E. Strongly disagree

22. In diagnosing Long COVID patients it is important to assess <u>neurological/brain</u> symptoms <u>including</u> sleep disruption, headache, seizures, cognitive dysfunction, mood changes, sensitivity to stimuli of the senses, hearing, vertigo, loss of smell/taste, dry eyes or

mouth, temperature dysregulation, paresthesias, changes in sweating, syncope, tremor/internal vibrations, hallucinations, depression and anxiety.

A. Strongly agree
B. Agree
C. Neither agree nor disagree
D. Disagree
E. Strongly disagree

23. In diagnosing Long COVID patients it is important to assess cardiorespiratory symptoms including dizziness on standing, palpitations, chest pain, SOB, cough, wheezing, and tachycardia with modest exertion.

A. Strongly agree
B. Agree
C. Neither agree nor disagree
D. Disagree
E. Strongly disagree

24. In diagnosing Long COVID patients it is important to assess vascular symptoms including limb pain or heaviness, nail changes, Raynaud's syndrome, hair loss, blotchy skin, vascular rash, and COVID toes.

A. Strongly agree
B. Agree
C. Neither agree nor disagree
D. Disagree
E. Strongly disagree

25. In diagnosing Long COVID patients it is important to assess gastrointestinal symptoms including changes in bowel habit, abdominal pain, nausea, bloating, food regurgitation or vomiting, new food intolerances, and changes in weight.

A. Strongly agree
B. Agree
C. Neither agree nor disagree
D. Disagree
E. Strongly disagree

26. In diagnosing Long COVID patients it is important to assess allergy symptoms including hives, anaphylaxis, new onset or worsening of existing allergies, dermatographism, nasal congestion or rhinorrhea, atopy, rashes, diarrhea, and joint pain.

A. Strongly agree
B. Agree
C. Neither agree nor disagree
D. Disagree
E. Strongly disagree

27. In diagnosing Long COVID patients it is important to assess genitourinary symptoms including sexual dysfunction, menstrual changes, loss of libido, urinary frequency, dysuria, and problems with bladder emptying.

A. Strongly agree
B. Agree
C. Neither agree nor disagree
D. Disagree
E. Strongly disagree

28. In diagnosing Long COVID patients it is important to screen for orthostatic intolerance and dysautonomia, including postural orthostatic tachycardia syndrome (POTS).

A. Strongly agree
B. Agree
C. Neither agree nor disagree
D. Disagree
E. Strongly disagree

29. In diagnosing Long COVID patients it is important to assess endocrine symptoms, for example weight loss/gain, tremors, polyuria/polydipsia (diabetes), premature menopause.

A. Strongly agree
B. Agree
C. Neither agree nor disagree
D. Disagree
E. Strongly disagree

30. In assessing Long COVID patients it is important to use imaging studies for persistent and unexplained symptoms

A. Strongly agree

B. Agree
C. Neither agree nor disagree
D. Disagree
E. Strongly disagree

31. Evaluation of all aspects of Long COVID should account for the fluctuating nature of many symptoms and recognise the need for repeated measurements to capture clinical findings (e.g. autonomic dysfunction, cognitive difficulties).
A. Strongly agree
B. Agree
C. Neither agree nor disagree
D. Disagree
E. Strongly disagree

32. In clinical assessment of Long COVID, it is important to evaluate for sleep disturbances.
A. Strongly agree
B. Agree
C. Neither agree nor disagree
D. Disagree
E. Strongly disagree

33. In clinical assessment of Long COVID, it is important to perform cardiopulmonary exercise testing in long covid to evaluate respiratory capacity,
A. Strongly agree
B. Agree
C. Neither agree nor disagree
D. Disagree
E. Strongly disagree

34. Do you have any comments or additional topics to be considered in the diagnosis or diagnostic tools for Long COVID?
Comment: ____

<u>IV. Treatment of Long COVID</u>
Treatment considerations

35. Long COVID biomarkers are important for <u>treatment</u> of the disease, despite its complexity.
A. Strongly agree
B. Agree
C. Neither agree nor disagree
D. Disagree
E. Strongly disagree

36. Multidisciplinary teams (MDTs) are a useful model for care of Long COVID.
A. Strongly agree
B. Agree
C. Neither agree nor disagree
D. Disagree
E. Strongly disagree

37. In treating Long COVID it is important to consider treatment of orthostatic intolerance and dysautonomia, including postural orthostatic tachycardia syndrome (POTS).
A. Strongly agree
B. Agree
C. Neither agree nor disagree
D. Disagree
E. Strongly disagree

38. In treating Long COVID it is important to consider treatment of abnormal clotting pathology.
A. Strongly agree
B. Agree
C. Neither agree nor disagree
D. Disagree
E. Strongly disagree

39. In treating Long COVID it is important to consider treatment of mast cell activation syndrome.
A. Strongly agree
B. Agree

C. Neither agree nor disagree
D. Disagree
E. Strongly disagree

40. In treating Long COVID it is important to consider treatment of SARS-CoV-2 viral persistence with antiviral medication.
A. Strongly agree
B. Agree
C. Neither agree nor disagree
D. Disagree
E. Strongly disagree

41. In treating Long COVID it is important to consider treatment of resting tachycardia.
A. Strongly agree
B. Agree
C. Neither agree nor disagree
D. Disagree
E. Strongly disagree

42. In treating Long COVID it is important to consider treatment of myocarditis/pericarditis.
A. Strongly agree
B. Agree
C. Neither agree nor disagree
D. Disagree
E. Strongly disagree

43. In treating Long COVID it is important to consider treatment of gut dysbiosis.
A. Strongly agree
B. Agree
C. Neither agree nor disagree
D. Disagree
E. Strongly disagree

44. In treating Long COVID is important to treat sleep disturbances
A. Strongly agree
B. Agree
C. Neither agree nor disagree
D. Disagree
E. Strongly disagree

45. In treating Long COVID it is important to consider treatment of newly identified diabetes and/or dyslipidemia, or consider modifying the treatment of pre-existing diabetes and/or dyslipidemia.
A. Strongly agree
B. Agree
C. Neither agree nor disagree
D. Disagree
E. Strongly disagree

46. In treating Long COVID it is important to consider treatment of newly identified pain, or consider modifying treatment of pre-existing pain.
A. Strongly agree
B. Agree
C. Neither agree nor disagree
D. Disagree
E. Strongly disagree

47. In treating Long COVID it is important to consider treatment of newly identified blood pressure abnormalities, or consider modifying treatment of pre-existing abnormalities.
A. Strongly agree
B. Agree
C. Neither agree nor disagree
D. Disagree
E. Strongly disagree

Treatment approaches

48. Treatments should be tailored to the history and clinical examination

A. Strongly agree
B. Agree
C. Neither agree nor disagree
D. Disagree
E. Strongly disagree

49. Cardiac or respiratory pathology should be ruled out before prescribing graded exercise therapy.
A. Strongly agree
B. Agree
C. Neither agree nor disagree
D. Disagree
E. Strongly disagree

50. Pain medications can be useful for treatment of pain in Long COVID.
A. Strongly agree
B. Agree
C. Neither agree nor disagree
D. Disagree
E. Strongly disagree

51. Antidepressant medications can be useful for treatment of Long COVID.
A. Strongly agree
B. Agree
C. Neither agree nor disagree
D. Disagree
E. Strongly disagree

52. In treating Long COVID it is important to consider use of anti-platelet drugs.
A. Strongly agree
B. Agree
C. Neither agree nor disagree
D. Disagree
E. Strongly disagree

53. Statins can be useful to treat Long COVID.
A. Strongly agree
B. Agree
C. Neither agree nor disagree
D. Disagree
E. Strongly disagree

54. Smell training, vitamin A (oil nasal drops), and steroid nasal rinses can be useful for treating olfactory dysfunction in Long COVID.
A. Strongly agree
B. Agree
C. Neither agree nor disagree
D. Disagree
E. Strongly disagree

55. Pharmacological agents like bupropion, venlafaxine, modafinil or amantadine can be useful for treating Long COVID symptoms.
A. Strongly agree
B. Agree
C. Neither agree nor disagree
D. Disagree
E. Strongly disagree

56. Selective serotonin reuptake inhibitors (SSRIs) can be useful for treating Long COVID patients.
A. Strongly agree
B. Agree
C. Neither agree nor disagree
D. Disagree
E. Strongly disagree

57. Nutritional and diet changes and nutritional supplements (such as vitamin B group) can be useful in treating some Long COVID patients.
A. Strongly agree
B. Agree
C. Neither agree nor disagree
D. Disagree
E. Strongly disagree

58. Yoga and Tai Chi can be useful in treating some Long COVID patients.
A. Strongly agree
B. Agree
C. Neither agree nor disagree
D. Disagree
E. Strongly disagree

59. Graded exercise can be useful in treating some Long COVID patients who do not have post exertional malaise (PEM) or post exertional symptom exacerbation (PESE).
A. Strongly agree
B. Agree
C. Neither agree nor disagree
D. Disagree
E. Strongly disagree

60. Vagus nerve therapies (eg. cold exposure, breathwork, mindfulness, compression wear, trauma release, vagus nerve stimulators) can be useful in treating some Long COVID patients.
A. Strongly agree
B. Agree
C. Neither agree nor disagree
D. Disagree
E. Strongly disagree

61. Pulmonary rehabilitation can be useful in treating some Long COVID patients.
A. Strongly agree
B. Agree
C. Neither agree nor disagree
D. Disagree
E. Strongly disagree

62. Melatonin can be useful in treating some Long COVID patients.
A. Strongly agree
B. Agree
C. Neither agree nor disagree
D. Disagree
E. Strongly disagree

63. Use of combined nattokinase and/or serrapeptase can be useful in treating some Long COVID patients.
A. Strongly agree
B. Agree
C. Neither agree nor disagree
D. Disagree
E. Strongly disagree

64. Hyperbaric oxygen therapy can be useful in treating some Long COVID patients.
A. Strongly agree
B. Agree
A. Neutral
C. Neither agree nor disagree
D. Disagree
E. Strongly disagree

65. Drugs that modulate the autonomic nervous system (e.g. ivabradine, beta blockers, midodrine) can be useful in treating some Long COVID patients.
A. Strongly agree
B. Agree
C. Neither agree nor disagree
D. Disagree

E. Strongly disagree

66. Anticoagulant drugs can be useful in treating some Long COVID patients.
A. Strongly agree
B. Agree
C. Neither agree nor disagree
D. Disagree
E. Strongly disagree

67. Paxlovid can be useful in treating some Long COVID patients.
A. Strongly agree
B. Agree
C. Neither agree nor disagree
D. Disagree
E. Strongly disagree

68. Duloxetine can be effective in treating peripheral neuropathy Long COVID.
A. Strongly agree
B. Agree
C. Neither agree nor disagree
D. Disagree
E. Strongly disagree

69. Drugs for treatment of gastroparesis and hyperacidity (e.g. proton pump inhibitors, H2-blockers) can be useful in treating Long COVID
A. Strongly agree
B. Agree
C. Neither agree nor disagree
D. Disagree
E. Strongly disagree

70. Do you have any comments or further suggestions in the treatment of Long COVID?
Comments: ____

Measuring the effect of treatment

71. The Symptom Burden Questionnaire for Long COVID (SBQ-LC) can be used to monitor the effect of treatment in patients with Long COVID.
A. Strongly agree
B. Agree
C. Neither agree nor disagree
D. Disagree
E. Strongly disagree

72. Cognitive screening tools (e.g. Montreal Cognitive Assessment or Mini-Mental State Examination) can be useful to measure the effects of Long COVID treatments.
A. Strongly agree
B. Agree
C. Neither agree nor disagree
D. Disagree
E. Strongly disagree

73. Fatigue assessment tools (e.g. Epworth Sleep Scale, Fatigue Severity Scale, etc.) can be useful to measure the effects of Long COVID treatments.
A. Strongly agree
B. Agree
C. Neither agree nor disagree
D. Disagree
E. Strongly disagree

74. Re-imaging can be useful to measure the effect of Long COVID treatment.
A. Strongly agree
B. Agree
C. Neither agree nor disagree
D. Disagree
E. Strongly disagree

75. Follow-up (repeat) examination with cognitive screening tools and physical examination can be useful to measure the effect of Long COVID treatment.
A. Strongly agree
B. Agree
C. Neither agree nor disagree
D. Disagree
E. Strongly disagree

76. For pediatric patients, back to school attendance, resuming sport, musical and other activities as normal might be a good measure of successful treatment.
A. Strongly agree
B. Agree
C. Neither agree nor disagree
D. Disagree
E. Strongly disagree

77. Repeated haemostatic tests for coagulopathy can be useful to measure the effect of Long COVID treatment.
A. Strongly agree
B. Agree
C. Neither agree nor disagree
D. Disagree
E. Strongly disagree

78. Repeating the cardiopulmonary exercise test (CPET) can be useful to measure the effect of Long COVID treatment.
A. Strongly agree
B. Agree
C. Neither agree nor disagree
D. Disagree
E. Strongly disagree

79. Do you have any comments or further suggestions to best evaluate treatment(s) for Long COVID?
Comments: ______________________________________

V. <u>Research areas for long COVID</u>

General research questions

80. Research into the pathomechanism(s) of Long COVID, including relevant organ systems, is of paramount importance to long-term treatment goals.
A. Strongly agree
B. Agree
C. Neither agree nor disagree
D. Disagree
E. Strongly disagree

81. Research into Long COVID should investigate the impact on previous or ongoing infections with other pathogens (e.g. co-infections, viral re-activation).
A. Strongly agree
B. Agree
C. Neither agree nor disagree
D. Disagree
E. Strongly disagree

82. Research into Long COVID should assess the impact of COVID-19 on increased susceptibility to infection in the post-COVID period.
A. Strongly agree
B. Agree
C. Neither agree nor disagree
D. Disagree
E. Strongly disagree

83. Research into treatments for Long COVID is vitally important
A. Strongly agree
B. Agree
C. Neither agree nor disagree
D. Disagree
E. Strongly disagree

84. Reducing transmission of SARS-CoV-2 will lower the incidence of Long COVID.
A. Strongly agree
B. Agree
C. Neither agree nor disagree
D. Disagree
E. Strongly disagree

85. Engineering to clean indoor air is an engineering issue that should be priotitised to lower the incidence and COVID and therefore Long COVID.
A. Strongly agree
B. Agree
C. Neither agree nor disagree
D. Disagree
E. Strongly disagree

86. Reducing transmission of SARS-CoV-2 will lower the incidence of Long COVID, but doing so is a political issue.
A. Strongly agree
B. Agree
C. Neither agree nor disagree
D. Disagree
E. Strongly disagree

87. If you like, please comment or make suggestions on general areas of research in Long COVID.
Comments: ____

Specific areas of Long COVID research

88. A major target area of research should be on the effects of COVID/Long COVID on the immune system.
A. Strongly agree
B. Agree
C. Neither agree nor disagree
D. Disagree
E. Strongly disagree

89. Viral persistence as a potential mechanism for Long COVID should be researched..
A. Strongly agree
B. Agree
C. Neither agree nor disagree
D. Disagree
E. Strongly disagree

90. A major target area of research should be on the effects of COVID/Long COVID on the neurological system.
A. Strongly agree
B. Agree
C. Neither agree nor disagree
D. Disagree
E. Strongly disagree

91. A major target area of research should be on the effects of COVID/Long COVID on the cardiac and vascular systems.
A. Strongly agree
B. Agree
C. Neither agree nor disagree
D. Disagree
E. Strongly disagree

92. A major target area of research should be on the effects of COVID/Long COVID on autoimmunity.
A. Strongly agree
B. Agree
C. Neither agree nor disagree
D. Disagree
E. Strongly disagree

93. A major target area of research should be on the effects of COVID/Long COVID on the respiratory system.
A. Strongly agree
B. Agree

C. Neither agree nor disagree
D. Disagree
E. Strongly disagree

94. A major target area of research should be on the effects of SARS-CoV-2 reinfections on COVID/Long COVID.
A. Strongly agree
B. Agree
C. Neither agree nor disagree
D. Disagree
E. Strongly disagree

95. A major target area of research should be on the effects of antivirals on COVID/Long COVID.
A. Strongly agree
B. Agree
C. Neither agree nor disagree
D. Disagree
E. Strongly disagree

96. A major target area of research should be on the effects of COVID/Long COVID in children.
A. Strongly agree
B. Agree
C. Neither agree nor disagree
D. Disagree
E. Strongly disagree

97. A major target area of research should be on the effects of COVID/Long COVID on sleep.
A. Strongly agree
B. Agree
C. Neither agree nor disagree
D. Disagree
E. Strongly disagree

98. A major target area of research should be on the effects of COVID/Long COVID on viral reactivation oncogenesis.
A. Strongly agree
B. Agree
C. Neither agree nor disagree
D. Disagree
E. Strongly disagree

99. A major target area of research should be on the effects of COVID/Long COVID on the autonomic nervous system, including the vagus nerve.
A. Strongly agree
B. Agree
C. Neither agree nor disagree
D. Disagree
E. Strongly disagree

100. A major target area of research should be on the relationship between COVID/Long COVID and oral health including the oral microbiome and periodontal disease.
A. Strongly agree
B. Agree
C. Neither agree nor disagree
D. Disagree
E. Strongly disagree

101. A major target area of research should be on the effects of COVID/Long COVID on glucose and lipid metabolism.
A. Strongly agree
B. Agree
C. Neither agree nor disagree
D. Disagree
E. Strongly disagree

102. A major target area of research should be on the effects of COVID/Long COVID on acute and prolonged states of inflammation.
A. Strongly agree
B. Agree
C. Neither agree nor disagree

D. Disagree
E. Strongly disagree

103. All systems in the body need to be considered in the research of Long COVID.
A. Strongly agree
B. Agree
C. Neither agree nor disagree
D. Disagree
E. Strongly disagree

104. If you like, please comment or make suggestions on specific areas of research in Long COVID.
Comments: ____

Organ and body damage

105. Damage to the liver, pancreas, kidneys, and/or skin might occur in COVID/Long COVID but may not always appear with initial symptoms or within the first few months.
A. Strongly agree
B. Agree
C. Neither agree nor disagree
D. Disagree
E. Strongly disagree

106. Damage to the nervous system might be incurred by COVID/Long COVID but does not always appear with initial symptoms.
A. Strongly agree
B. Agree
C. Neither agree nor disagree
D. Disagree
E. Strongly disagree

107. Damage to the patient's endothelium and/or microvasculature might occur in COVID/Long COVID but does not always appear with initial symptoms.
A. Strongly agree
B. Agree
C. Neither agree nor disagree
D. Disagree
E. Strongly disagree

108. Covid-19 may cause direct damage to cardiomyocytes. This may occur in the absence of a rise in cardiac biomarkers such as troponin.
A. Strongly agree
B. Agree
C. Neither agree nor disagree
D. Disagree
E. Strongly disagree

109. Covid-19 may result in reduced pulmonary function or pulmonary fibrosis.
A. Strongly agree
B. Agree
C. Neither agree nor disagree
D. Disagree
E. Strongly disagree

110. Covid-19 may be associated with a higher risk of dementia or acceleration of dementia.
A. Strongly agree
B. Agree
C. Neither agree nor disagree
D. Disagree
E. Strongly disagree

111. Covid-19 may cause type 1 or type 2 diabetes
A. Strongly agree
B. Agree
C. Neither agree nor disagree
D. Disagree
E. Strongly disagree

112. If you like, please comment or make suggestions on research in Long COVID related to organ or body damage.
Comments: ____

Children and young people

113. The impact of Long COVID on children's attendance of and performance in school should be researched.
A. Strongly agree
B. Agree
C. Neither agree nor disagree
D. Disagree
E. Strongly disagree

114. The effect of vaccination and boosters on Long Covid incidence and severity in children should be researched.
A. Strongly agree
B. Agree
C. Neither agree nor disagree
D. Disagree
E. Strongly disagree

115. The impact of Long Covid on the developing brain in children should be researched.
A. Strongly agree
B. Agree
C. Neither agree nor disagree
D. Disagree
E. Strongly disagree

116. The impact of Long Covid on the mental health of children should be researched.
A. Strongly agree
B. Agree
C. Neither agree nor disagree
D. Disagree
E. Strongly disagree

117. The impact of Long Covid on new onset diabetes in children should be researched.
A. Strongly agree
B. Agree
C. Neither agree nor disagree
D. Disagree
E. Strongly disagree

118. The impact of Long Covid on the development of narcolepsy and sleep disordered breathing in children should be researched.
A. Strongly agree
B. Agree
C. Neither agree nor disagree
D. Disagree
E. Strongly disagree

119. The impact of Long Covid on the immune systems of children should be researched.
A. Strongly agree
B. Agree
C. Neither agree nor disagree
D. Disagree
E. Strongly disagree

120. The long-term impact of Long Covid on children should be researched.
A. Strongly agree
B. Agree
C. Neither agree nor disagree
D. Disagree
E. Strongly disagree

121. If you like, please comment or make suggestions on research concerning Long COVID in children and young people.
Comments: ____

Vaccination and Long COVID

122. Long COVID-like symptoms can occur following vaccination.

A. Strongly agree
B. Agree
C. Neither agree nor disagree
D. Disagree
E. Strongly disagree

123. Vaccination can cause ongoing symptoms in some people, and this should be researched.
A. Strongly agree
B. Agree
C. Neither agree nor disagree
D. Disagree
E. Strongly disagree

124. Long COVID can improve with vaccination
A. Strongly agree
B. Agree
C. Neither agree nor disagree
D. Disagree
E. Strongly disagree

125. Where vaccination has led to or impacted Long COVID symptoms this should be carefully researched and patient risks from different vaccines identified to inform guidelines
A. Strongly agree
B. Agree
C. Neither agree nor disagree
D. Disagree
E. Strongly disagree

126. Where vaccination has led to vaccine injury or impacted Long COVID symptoms this should be treated
A. Strongly agree
B. Agree
C. Neither agree nor disagree
D. Disagree
E. Strongly disagree

127. If you like, please comment or make suggestions on research concerning Long COVID and vaccination.
Comments: ____

VI. *Who should fund Long COVID research?*

128. Governments have a responsibility to contribute to the funding of Long COVID research.
A. Strongly agree
B. Agree
C. Neither agree nor disagree
D. Disagree
E. Strongly disagree

129. Corporate entities have a responsibility to contribute to the funding of Long COVID research.
A. Strongly agree
B. Agree
C. Neither agree nor disagree
D. Disagree
E. Strongly disagree

130. All funding towards Long COVID research should be funded in a transparent manner.
A. Strongly agree
B. Agree
C. Neither agree nor disagree
D. Disagree
E. Strongly disagree

131. A major target for research should be the economic and societal impacts of Long COVID.
A. Strongly agree
B. Agree

C. Neither agree nor disagree
D. Disagree
E. Strongly disagree

132. If you like, please comment or make suggestions on research concerning Long COVID funding and economic impacts.
Comments: ____

Table S6. List of statements for round 3 of the survey

WHN Long Covid Delphi Consensus Round 3 (statements were given a choice of Strongly agree, Agree, Neither agree nor disagree, Disagree, Strongly disagree

Q1. Your specific responses will remain confidential. Your name and affiliation will only be used to identify you as a respondent to the executive committee. When the manuscript is written, you will be given a chance to comment and will need to consent to being a coauthor at that time. You can read the terms of the Conditions of Consent here. Do you agree with the Consent Information Sheet? If you agree to participate, please continue the e-survey. If you do not wish to participate, please exit the survey.
Q2. Because of the heterogeneity of health problems that occur after COVID-19, the term "Post COVID Conditions" should be used instead of "Long COVID," even if SARS-CoV2 viral/antigen persistence is a potential cause of some of those health problems.

Q3. As Long COVID is a broad and inclusive term, it is valuable to create subcategories based on phenotype/endotype.
Q4. Acknowledging that many clinical phenotypes of Long COVID align with established syndromes such as post-intensive care syndrome, ME/CFS, and POTS, it is important to recognize that these conditions may have distinct pathophysiologic mechanisms.

Q5. The term Long COVID should be used for those that have persistent or relapsing and remitting functional impairment.
Q6. Long COVID is triggered by SARS-CoV2 infection; the resulting pathophysiology may include immune-mediated multi-organ inflammatory changes, endothelial dysfunction, hypercoagulability, microclotting, and downstream effects on mitochondrial dysfunction.
Q7. Long COVID best describes the persistence of symptoms that started with COVID-19 and have failed to resolve 8 weeks after the onset of the illness.
Q8. Long COVID includes any new symptoms with onset after acute COVID-19, whether those symptoms appear during the acute infection or have their onset within a period of 12 weeks after the acute infection and persist for at least 8 weeks.
Q9. Long COVID includes the exacerbation of pre-existing symptoms or a pre-existing medical condition for at least 8-12 weeks after COVID-19.
Q10. Long COVID is characterized by the persistence of symptoms and signs for at least 3 months after the onset of initial symptoms or a recurrence within 2 months after the resolution of acute COVID. The evolving nature of symptoms during the course of the illness contributes to a reduction in functional capacity and overall health compared to the pre-infection state, potentially exacerbating pre-existing health conditions.
Q11. Long COVID includes impairment in any area of function e.g. physical or cognitive, that follows acute COVID-19 and persists for at least 8-12 weeks, independent of symptoms.
Q12. Long COVID includes abnormalities in laboratory tests (i.e. liver or kidney function tests) that appear during or within 3 months of acute COVID-19 and persist for at least 8-12 weeks, whether or not there are symptoms or functional disabilities.
Q13. Long COVID includes new onset of a specific disease entity i.e. with a pre-pandemic ICD code, that starts within 3 months after acute COVID-19. Examples include diabetes, hypertension, migraine headache, and autoimmune or allergic diseases.
Q14. The criteria for diagnosing Long COVID in adults should be the same as the criteria for diagnosing Long COVID in children.
Q15. Long COVID is evident in young individuals with a documented history of SARS-CoV-2 infection, displaying at least one enduring physical, cognitive, or neuropsychiatric symptom that persists for a minimum of 8-12 weeks after the initial infection, where other causes have been excluded.
Q16. It is important to recognize that Long COVID is an umbrella term encompassing several different disorders including e.g. dysautonomia, neuroinflammation, endothelial dysfunction, hypercoagulation, impaired fibrinolysis, mast cell disorders, and mitochondrial dysfunction. It is therefore important to establish a minimum Long COVID diagnostic workup for these conditions.
Q17. Long COVID is not a singular disease entity; it comprises distinct yet related virally triggered host response pathways. Proper diagnosis is crucial, considering conditions like virally induced POTS, MCAS, acquired vascular phenotype, post-COVID neuroinflammation, and post-COVID myocarditis. Recognizing each diagnosis separately is essential as treatments differ with each.

Q18. It is important that clinicians realise that most standard screening tests will come back normal and specific tests are needed for diagnosis of various pathologies in Long COVID.
Q19. The diagnosis of Long COVID requires that the person have laboratory confirmation of COVID-19 infection during a period of 4-8 weeks prior to the onset of symptoms.
Q20. The diagnosis of Long COVID does not require that the person have laboratory confirmation of COVID-19 infection during a period of 4-8 weeks prior to the onset of symptoms.
Q21. There is a need to educate all health care workers about the possible complications following SARS-CoV-2 infection and to ensure that patients are listened to, appropriately investigated and supported.

Q22. Diagnostic investigations should be tailored to the symptoms of each patient.
Q23. Clinically validated Long COVID-specific biomarkers, when available, will play a role in the diagnosis of Long COVID, despite the complexity of the disease.
Q24. Novel tests for microclot formation need to be further validated and integrated into clinical use as potential vascular biomarkers of Long COVID.
Q25. Emergent biomarkers of neuroaxonal or glial fibrillary damage, such as NfL and GFAP, can be important in diagnosing Long COVID.
Q26. It is important to ask Long COVID patients about changes in their ability to perform activities of daily living (ADLs) to understand disease severity.
Q27. It is essential to screen for appropriate categories of cardiovascular disease relevant to a patient's clinical presentation in the workup of Long COVID.
Q28. Assessment of endothelial function and platelet hyperactivation should be routinely carried out in Long COVID patients as part of the workup and ongoing management when these tests become available to clinicians in the future.
Q29. In diagnosing Long COVID, testing for thrombophilias or indicators for increased clotting risk (i.e. Factor V Leiden, anti-phospholipid antibodies, homocysteine, prothrombin, hemochromatosis testing, etc.) can be helpful.
Q30. In the clinical and diagnostic assessment of Long COVID pathophysiology, cardiopulmonary exercise testing (CPET) can be used to evaluate cardiorespiratory function and functional capacity in patients for whom the testing is safe and appropriate.
Q31. Evaluation for neuropsychiatric manifestations, such as anxiety, depression, sleep disturbance, cognitive disturbance, and/or ADHD should be part of a comprehensive assessment of Long COVID patients.
Q32. It is important to evaluate for symptoms of perimenopause, premature onset menopause and worsening of existing menopause in females as part of Long COVID evaluation, as there is some data showing that COVID-19 can trigger hormonal changes.
Q33. Careful assessment for autoimmune, cardiorespiratory, endocrine, and other post-COVID complications across body systems is an important part of the management of Long COVID.

Q34. Screening for iron deficiency can be useful as part of a comprehensive assessment in Long COVID.
Q35. In treating Long COVID it is important to consider treatment of SARS-CoV-2 viral persistence with treatments that have antiviral effects.
Q36. Non-opioid pain medications can be useful for treatment of e.g. small fibre neuropathy pain and headache in Long COVID.
Q37. Psychological therapies can be useful in supporting the mental health of those with Long COVID in conjunction with treatments that target the pathophysiology.
Q38. Antidepressants can be useful in supporting the mental health of those with Long COVID where appropriate, and in conjunction with other treatments that target the pathophysiology.

Q39. Therapies directed at endothelialitis or endothelial injury are useful in treating Long COVID.
Q40. Pharmacological agents that promote wakefulness and/or are CNS stimulants can be useful for managing fatigue, hypersomnolence, and cognitive dysfunction in Long COVID patients.
Q41. Nutritional and diet changes and nutritional supplements (such as B vitamins and probiotics) can be useful in managing symptoms in some patients with Long COVID.

Q42. Yoga and Tai Chi can be useful in managing symptoms in some patients with Long COVID.

Q43. Treatments that target Vagus nerve dysfunction can be useful in managing Long COVID symptoms.
Q44. Therapies that stimulate the Vagus nerve and/or promote parasympathetic activation of the autonomic nervous system (e.g. mindfulness, breathwork, cold water exposure, cryotherapy, trauma release, cranial osteopathy, acupuncture) can be useful in treating patients with Long COVID.

Q45. Electronic devices that stimulate the Vagus nerve can be useful in treating patients with Long COVID.
Q46. Wearable devices that track heart rate variability (HRV) are useful to guide the pacing of activity and exertion in Long COVID.
Q47. Pulmonary rehabilitation (an established exercise training and education programme for people with structural lung disease) is not indicated for the majority of people with Long COVID related breathlessness.

Q48. Melatonin can be useful in treating Long COVID related insomnia.

Q49. Melatonin can be useful as an anti-inflammatory and antioxidative agent in patients with Long COVID.
Q50. Hyperbaric oxygen therapy can be useful in treating a subgroup of Long COVID patients with cognitive dysfunction and evidence of end-organ hypoxia e.g. decreased left ventricular function.
Q51. Anticoagulant and antiplatelet drugs can be used to treat a sub-population of patients with Long COVID, as long as appropriate diagnostic tools for thrombotic endothelialitis are available and treatment is overseen by an experienced clinician.
Q52. Drugs for treatment of gastroparesis e.g. metoclopramide, domperidone, pyridostigmine, can be useful in treating Long COVID patients with dysautonomia.
Q53. Cognitive screening tools such as the Montreal Cognitive Assessment (MOCA) or the Mini Mental State Exam (MMSE) may not be appropriate for testing of cognitive disturbance in patients with Long COVID. More comprehensive cognitive testing, sometimes performed on more than one occasion, may be required to detect & assess the severity of cognitive dysfunction in Long COVID patients.
Q54. Re-imaging for specific Long COVID complications such as pulmonary embolism, myocarditis, and heart failure can be useful to monitor the response to treatment in patients with Long COVID.
Q55. If available, repeat measurement of markers of endothelial dysfunction, platelet hyperactivation and abnormal clotting physiology (such as vWF, sCD40 ligand, VEGF & microclot detection) can be useful to track the effect of some Long COVID treatments.
Q56. If determined safe and appropriate following detailed screening for post-exertional malaise (PEM), repeat cardiopulmonary exercise testing (CPET) can be important to monitor changes in VO2 max and anaerobic threshold and to measure the effectiveness of treatments (including rehabilitation programmes) in Long COVID.
Q57. Reducing transmission of SARS-CoV-2 will lower the incidence of COVID-19 reinfections in individuals and thus decrease the incidence of Long COVID.
Q58. Cleaning indoor air is an issue that should be prioritised to lower the incidence of acute COVID-19 infections and, therefore Long COVID.
Q59. An international task force should be formed to develop a consensus on Long COVID research priorities and facilitate/encourage global collaborative efforts and data sharing.

Q60. A major medical/scientific research goal should be establishing pathogenesis of Long COVID.
Q61. Research should look at the future societal and economic impacts of SARS-CoV-2. This research should assess and include the potential rise in health, social and economic burdens of other chronic diseases triggered or worsened by SARS-COV-2.

Q62. A target area of research should be on the effects of antivirals on COVID/Long COVID.

Q63. A target area of research should be on the effects of COVID/Long COVID on sleep.

Q64. A major target area of research should be on the immune dysfunction associated with COVID/Long COVID.

Q65. A target area of research should be on the effects of COVID/Long COVID on oncogenesis.

Q66. The relationship between Long COVID and the gut microbiome/dysbiosis is an important area to research.
Q67. Given the link between poor oral health & adverse effects from COVID-19, a target area of research should be on the relationship between COVID/Long COVID and oral health, including the oral microbiome and periodontal disease.
Q68. Evaluation of gut / nasal / oral / vaginal microbiome dysbiosis, with a particular focus on acetaldehyde producing vs. metabolising species, should be investigated as a diagnostic tool for Long COVID.
Q69. Mechanisms of and treatments for post-exertional malaise/post exertion symptom exacerbation (PEM/PESE) in Long COVID is an important area of research.

Q70. Investigating autonomic dysfunction in Long COVID is an important area of research.
Q71. Development of evidence-based treatment protocols for endothelial dysfunction and coagulopathy in COVID and Long COVID is an important area of research.
Q72. Understanding factors that exist pre-infection and during acute COVID-19 infection that predispose to development of Long COVID is an important area of research.
Q73. A target area of research should be on the effects COVID/Long COVID on mitochondrial function as well as cellular metabolism and senescence.
Q74. There needs to be more research into the relationship between Long COVID and autoimmunity as well as investigation into novel biomarkers such as antibodies to G protein-coupled receptors (GPCRs).

Q75. Markers of neutrophil extracellular trap (NET) formation should be investigated in Long COVID.

Q76. Markers of mitochondrial dysfunction should be investigated in Long COVID.

Q77. Research into the pathophysiology of Long COVID should include nutritional and metabolic status, e.g. trace elements, amino acids, organic acids, intracellular minerals, trace elements, and electrolyte stores, as well as energy metabolites.
Q78. Multi-organ damage affecting the liver, pancreas, kidneys, and/or skin can occur in COVID/Long COVID but may not always appear with initial symptoms or within the first few months.

Q79. Reinfections can increase the risk of multi-organ damage from COVID/Long COVID.
Q80. Clinical research has already demonstrated that COVID-19 infection can trigger or accelerate neurodegenerative diseases like dementia, Parkinson's disease, and motor-neuron diseases.
Q81. The impacts of COVID-19 infection on children should be a research priority (e.g. prevention of transmission in schools, long-term impacts of infections, impacts on learning/development, etc).

Q82. There is a need to study the impact of repeated COVID-19 infections on Long COVID in both children and adults.
Q83. Studying the effects of acute COVID-19 on children's physical and cognitive performance and how it might lead to Long COVID should be prioritised as a research area.
Q84. Research into the physiological effects of Long COVID in children, including thrombotic endothelialitis (e.g. endothelial damage, activated platelets and microclots), viral persistence, and gastrointestinal impacts should be a priority.
Q85. It is important to study the psychological and mental health impacts of Long COVID on children in addition to the physiological effects (resulting in pathophysiological manifestations), especially with repeated COVID-19 infections.
Q86. The impact of repeated SARS-CoV2 infections on children's behaviour, cognition, concentration, and mental health should be an area of research priority.
Q87. Investigating the effects of Long COVID on development during puberty in adolescents should be an area of research priority.
Q88. Investigating the effects of Long COVID on the academic achievement of adolescents should be an area of research priority.
Q89. The similarities and differences in viral persistence between adults and children, and the phenomenon of viral reactivation in both adults and children should be researched.
Q90. A research priority should be to investigate why some children develop pediatric acute-onset neuropsychiatric syndrome (PANS) or MIS-C after acute COVID-19 infection and others do not.
Q91. Investigating the impact of COVID-19 vaccinations and boosters on the incidence and severity of Long COVID in children who have had COVID-19 infection should be a priority.
Q92. Assessing the social and educational consequences of Long COVID, including school attendance and performance, and social interaction challenges should be a priority.
Q93. Exploring a comprehensive approach to the treatment of Long COVID in children, encompassing biological, psychological, social, and ecological factors should be a priority.

Q94. SARS-CoV-2 vaccination can trigger a syndrome similar to Long COVID in some individuals.

Q95. Vaccination can reduce the risk of Long COVID but does not prevent it.

Q96. Research should focus on developing vaccines for SARS-CoV-2 that are less likely to cause serious adverse effects.
Q97. Research into the mechanisms of vaccine-induced Long COVID-type symptoms will help in the development of safer vaccines for everyone.
Q98. An area for research should be which vaccines are least likely to worsen symptoms in patients with pre-existing Long COVID.
Q99. Research needs to look at which Long COVID patients may be at increased risk of adverse effects following COVID vaccination (such as those with ME/CFS) so that individualised tailored decisions can be made.

Q100. Funding should be allocated to research Long COVID AND its impact on society.

Q101. Health insurance companies should support research into Long COVID and assume a more active role in the solution.
Q102. Health policies that encourage children to attend school while actively infected with COVID are likely to further increase absences rather than aid attendance rates and may result in increased Long COVID in Children.
Q103. Health policies that encourage children to attend school while actively infected with COVID are likely to increase Long COVID in parents.

Q104. Schools should be required to offer remote learning and other educational aids for children with Long COVID.
Q105. Certain professions (e.g. airline pilots, medical professionals, professional drivers) should be routinely tested for Long COVID-induced cognitive impairment after infection and reinfection if validated tests become clinically available.
